# New Thio-Compounds and Monoterpenes With Anti-inflammatory Activities From the Fungus *Aspergillus* sp. CYH26

**DOI:** 10.3389/fmicb.2021.668938

**Published:** 2021-03-25

**Authors:** Guojun Pan, Yanling Li, Xinyu Che, Dan Tian, Wenjie Han, Zimin Wang, Yanfen Zhao, Shuang Ren, Yiru Xu, Gangping Hao, Mengfei Guo, Na Xiao, Fandong Kong

**Affiliations:** ^1^College of Life Sciences, Shandong First Medical University, Shandong Academy of Medical Sciences, Tai’an, China; ^2^State Key Laboratory of Crop Biology, College of Agronomy, Shandong Agriculture University, Tai’an, China; ^3^Key Laboratory of Chemistry and Engineering of Forest Products, State Ethnic Affairs Commission, Guangxi Key Laboratory of Chemistry and Engineering of Forest Products, Guangxi Collaborative Innovation Center for Chemistry and Engineering of Forest Products, School of Chemistry and Chemical Engineering, Guangxi University for Nationalities, Nanning, China; ^4^State Key Laboratory of Natural Medicines, China Pharmaceutical University, Nanjing, China

**Keywords:** *Aspergillus* sp. CYH26, thio-compounds, monoterpenes, anti-inflammatory activity, fungus

## Abstract

Two new thio-compounds named aspergerthinol A and B (**1** and **2**) and two new monoterpenes named aspergerthinacids A and B (**3** and **4**) were isolated from the fungus *Aspergillus* sp. CYH26 from the rhizosphere soil of *Cynanchum bungei* Decne. The structures of compounds were elucidated by spectroscopic data and quantum NMR and ECD calculations. Compounds **1** and **2** represented a new family of sulfur containing natural products with a 3-methyl-4*H*-cyclopenta[b]thiophen-4-one skeleton. Compounds **1–4** showed inhibitory activities against nitric oxide (NO) with IC_50_ values of 38.0, 19.8, 46.3, and 56.6 μM, respectively.

## Introduction

**FIGURE 1:**
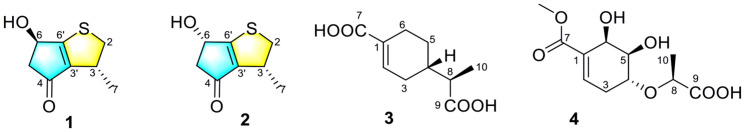
The chemical structures of compounds **1**–**4**.

Early inflammatory response is generally to eliminate the harmful stimulation condition (Cameron and Landreth, 2010). However, low-grade, chronic inflammation response plays a critical role in the pathological process of many diseases, such as arthritis, cancer, type 2 diabetes, and autoimmune diseases (Chen et al., 2017). Nitric oxide (NO) as a pro-inflammatory mediator is critical in the secretion of proinflammatory cytokines such as IL-6, TNF-α (Sharma et al., 2007). Thus, suppressing the production of NO could be a potential therapeutic method in the prevention of various diseases induced by excessive inflammation. Microorganism, including bacteria, actinomycetes, and fungi, have been proven to be a prolific source of structurally new and biologically active natural products (Qadri et al., 2013). The secondary metabolites obtained from fungi have gained considerable attention, as they contribute most of the new natural products from microorganism and many compounds from fungi possess unique structure with potent biological activity (Keller, 2019). In our ongoing search for new bioactive metabolites from fungi (Kong et al., 2017, 2019), the secondary metabolites produced by the soil fungus *Aspergillus* sp. CYH26 isolated from the rhizosphere soil of *Cynanchum bungei* Decne. from Mount Tai, China, were investigated, which resulted in the isolation and identification of two new thio compounds named aspergerthinol A and B (**1** and **2**) and two new monoterpenes named aspergerthinacids A and B (**3** and **4**) (Figure 1). All of the compounds could suppress the over-production of NO without affecting the cell viability. Herein, we describe the isolation, structural elucidation, and bioactivities of compounds **1**–**4**.

## Materials and Methods

### General Experimental Procedures

Optical rotations were measured on a JASCO P-1020 digital polarimeter, and UV spectra were measured on a Beckman DU 640 spectrophotometer. ECD data were collected using a JASCO J-715 spectropolarimeter. NMR spectra were recorded on a Bruckmercury Plus-400 or a JNM-ECZR-500 spectrometers with TMS as an internal standard. HRESIMS spectra were recorded with a Micromass Autospec -Uitima- TOF, API QSTAR Pulsar 1, or Waters Autospec Premier spectrometer. Semi-preparative HPLC was carried out using an ODS column (YMC-pack ODS-A, 10 × 250 mm, 5 μm, 4 mL/min). Thin layer chromatography (TLC) and column chromatography (CC) were performed on plates precoated with silica gel GF_254_ (10–40 μm, Yantai Jiangyou Silicone Development Co., Ltd.).

### Fungal Material and Fermentation

The fungus *Aspergillus* sp. CYH26 was isolated from the rhizosphere soil of *C. bungei* Decne., from Mount Tai, China in December 2018. After grinding, the sample (1.0 g) was diluted to 10^–2^ g/mL with sterile H_2_O, 100 μL of which was deposited on PDA (potato 200 g, glucose 20 g, agar 20 g per liter of tap water) plate and Bengal red medium (maltose 20 g, monosodium glutamate 10 g, glucose 10 g, yeast extract 3 g, corn pulp 1 g, mannitol 20 g, sodium chloride 0.3 g, potassium dihydrogen phosphate 0.5 g, agar 20 g per liter of tap water) plate containing chloramphenicol (200 μg/mL) as a bacterial inhibitor. A single colony was transferred onto another PDA plate and was identified according to its morphological characteristics and ITS gene sequences (GenBank accession No. MW578366, Supplementary Material). A reference culture of *Aspergillus* sp. CYH26 maintained at −80°C is deposited in our laboratory. The isolate was cultured on plates of PDA medium at 28°C for 4 days. Plugs of agar supporting mycelium growth were cut and transferred aseptically to 5 × 250 mL Erlenmeyer flasks each containing 100 mL of liquid medium (potato 200 g, glucose 20 g per liter of tap water) and cultured at 28°C at 150 RPM for 3 days. The seed liquid was inoculated aseptically into 50 × 1,000 mL Erlenmeyer flasks each containing rice medium (80 g rice, 100 mL tap water) at 1% inoculation amount and incubated at room temperature under static conditions for 35 days.

### Extraction and Isolation

The cultures (4 kg) were then extracted in to EtOAc (20 L) by soaking overnight. The extraction was repeated for three times. The combined EtOAc extracts were dried under vacuum to produce 22.6 g of extract. The EtOAc extract was subjected to a silica gel VLC column, eluting with a stepwise gradient of 0, 9, 11, 15, 20, 30, 50, and 100% EtOAc in petroleum ether (v/v), to give eight fractions (Fr. 1–8). Fraction 4 (3.4 g) was applied to ODS silica gel with gradient elution of MeOH-H_2_O (1:5, 2:3, 3:2, 4:1, and 1:0) to yield five subfractions (Fr. 4-1–Fr. 4-5). Fr. 4-2 (90 mg) was purified using semi-prep HPLC (isocratic system 50% MeOH/H_2_O, v/v) to give compounds **4** (*t*_R_ 6.0 min; 7.7 mg) and **3** (*t*_R_ 9.5 min; 11 mg). Fraction 5 (1.3 g) was applied to ODS silica gel with gradient elution of MeOH-H_2_O (1:5, 2:3, 3:2, 4:1, and 1:0) to yield four subfractions (Fr. 5-1–Fr. 5-5). Fr. 5-2 (71 mg) was further purified using semi-prep HPLC (isocratic system 40% MeOH/H_2_O, v/v) to give compounds **1** (*t*_R_ 5.3 min; 5.3 mg) and **2** (*t*_R_ 5.6 min; 4.1 mg).

*Aspergerthinol A (**1**)*: brown oils; [α]25 D −10 (*c* 0.1, MeOH); UV (MeOH) λ_max_ (log ε): 304 (2.85) nm; ECD (1.18 mM, MeOH) λ_max_ 207 (−5.31), 298 (−4.71), 325 (+3.68) nm. ^1^H and ^13^C NMR data, Table 1; HRESIMS *m/z* 169.0318 [M-H]^–^ (calcd for C_8_H_9_O_2_S, 169.0329).

**TABLE 1:** The ^1^H (400 MHz) and ^13^C NMR (100 MHz) Data of Compounds **1** and **2** in CD_3_OD.

**Position**	**1**		**2**	
	**δ_C_**	**δ_H_ (*J* in Hz)**	**δ_C_**	**δ_H_ (*J* in Hz)**
2	49.0, CH_2_	3.39, dd (11.5, 6.1)	49.0, CH_2_	3.39, dd (11.5, 5.1)
		4.02, dd (11.5, 9.2)		4.03, dd (11.5, 9.1)
3	36.5, CH	3.26, m	36.4, CH	3.27, m
3′	147.7, C		147.8, C	
4	197.6, C		197.5, C	
5	51.7, CH_2_	2.56, dd (18.0, 1.8)	51.7, CH_2_	2.55, dd (18.0, 1.9)
		3.05, dd (18.0, 6.2)		3.08, dd (18.0, 6.2)
6	67.0, CH	4.89, m	67.2, CH	4.91, m
6′	187.7, C		187.8, C	
7	18.1, CH_3_	1.25, d (6.8)	17.7, CH_3_	1.19, d (6.8)

*Aspergerthinol B (**2**)*: brown oils; [α]25 D −7 (*c* 0.1, MeOH); UV (MeOH) λ_max_ (log ε): 304 (2.84) nm; ECD (0.35 mM, MeOH) λ_max_ 217 (+ 5.63), 296 (+ 3.27), 323 (−3.31) nm. ^1^H and ^13^C NMR data, Table 1; HRESIMS *m/z* 169.0323 [M-H] ^–^ (calcd for C_8_H_9_O_2_S, 169.0329).

*Aspergerthinacid A (**3**)*: yellow powder; [α]25 D −46 (*c* 0.1, MeOH); UV (MeOH) λ_max_ (log ε): 218 (2.80) nm; ECD (1.2 mM, MeOH) λ_max_ 214 (−1.54) nm. ^1^H and ^13^C NMR data, Table 2; HRESIMS *m/z* 197.0811 [M-H] ^–^ (calcd for C_10_H_13_O_4_, 197.0819).

**TABLE 2:** The ^1^H (400 MHz) and ^13^C NMR (100 MHz) Data of Compounds **3** and **4** in CD_3_OD.

**Position**	**3**		**4**	
	**δ_C_**	**δ_H_ (*J* in Hz)**	**δ_C_**	**δ_H_ (*J* in Hz)**
1	131.4, C		129.8, C	
2	140.1, CH	6.95, m	139.4, CH	6.78, m
3	31.1, CH_2_	2.03, m	28.6, CH_2_	2.66, dddd (18.1, 4.7, 2.0, 2.0)
		2.25, m		2.29, dddd (18.1, 4.7, 1.6, 1.6)
4	37.1, CH	1.80, m	77.0, CH	3.79, ddd (7.1, 4.7, 4.7)
5	26.2, CH_2_	1.31, m	71.0, CH	3.86, dd (7.1, 4.5)
		1.92, m		
6	25.3, CH_2_	2.42, m	67.1, CH	4.40, m
		2.17, m		
7	170.7, C		168.6, C	
8	45.2, CH	2.32, overlap	74.8, CH	4.26, q (7.0)
9	179.8, C		176.0, C	
10	14.7, CH_3_	1.18, d (7.0)	19.5, CH_3_	1.34, d (6.9)

*Aspergerthinacid B (**4**)*: yellow powder; [α]25 D −168 (*c* 0.1, MeOH); UV (MeOH) λ_max_ (log ε): 213 (2.83) nm; ECD (3.7 mM, MeOH) λ_max_ 213 (−2.58) nm. ^1^H and ^13^C NMR data, Table 2; HRESIMS *m/z* 259.0824 [M-H] ^–^ (calcd for C_11_H_15_O_7_, 259.0823).

### Bioactivity Assay

Cell viability of the isolated compounds was detected using MTT assay. Mouse macrophages (RAW264.7 cells) obtained from the Type Culture Collection of the Chinese Academy of Sciences (Shanghai, China), were cultured in DMEM (Gibco, United States) with 10% FBS at 37°C in a 5% CO_2_ atmosphere. After that, cells were seeded in a 96-well plate (2 × 10^4^ cells/well) and pre-treated with different doses of compounds for 24 h at 37°C. After that, 10 μL of MTT was added to each well and incubated for another 4 h. The media and MTT were removed and 150 μL of dimethylsulfoxide (DMSO) was added. Following incubation for 0.5 h, the absorbance value was determined at 570 nm. For Determination of NO content, cells were seeded in a 96-well plate and pre-treated with different dose of compounds, and then stimulated with or without LPS (5 μg/mL) for another 24 h. The supernatants were analyzed for NO by commercial kit (Jiancheng, Nanjing, China). All tests were performed in triplicate. The NO inhibitory rate (%) was calculated as [1 − (A_drug_– A_blank_)/(A_control_– A_b__lank_)] × 100%. The IC_50_ values were calculated using prism software (GraphPad). The NO inhibitory rate (%) was calculated as [1 − (A_drug_–A_blank_)/(A_control_–A_blank_)] × 100%. The IC_50_ values were calculated using prism software (GraphPad).

## Results and Discussion

### Structure Elucidation of Compounds

**FIGURE 2:**
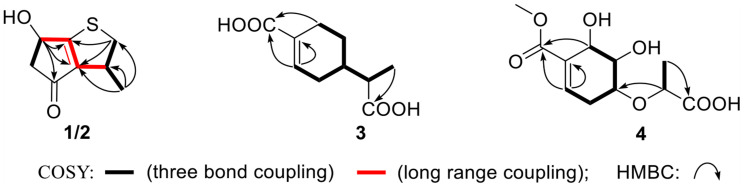
Selected HMBC and COSY correlations of **1**–**4**.

**FIGURE 3:**
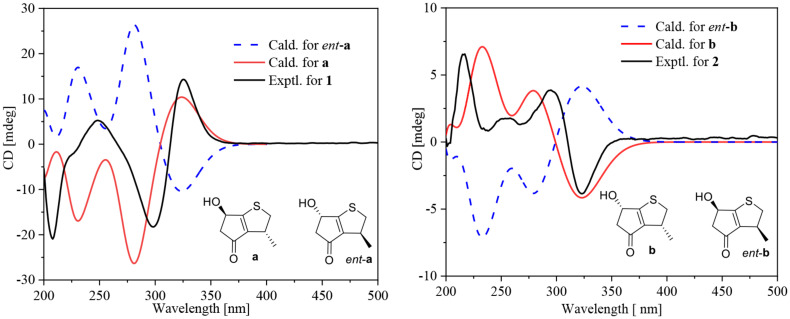
Experimental ECD curves for **1** and **2** and calculated ECD curves for the four possible isomers **a**, *ent*-**a**, **b**, and *ent*-**b**.

Compounds **1** and **2** were assigned the molecular formula C_10_H_8_O_2_S by HRESIMS. The double-bond equivalents of **1** and **2** were calculated to be four. The ^13^C and HSQC NMR spectra (Table 1) of **1** revealed a total of eight carbons including one ketone carbonyl, two olefinic non-protonated carbons, two sp^3^ methylenes with one heteroatom-bonded, two sp^3^ methines with one oxygenated, and one methyl. Detailed analysis of the COSY data (Figure 2) of **1** revealed the presence of two partial structures CH_3_-7/CH-3/CH_2_-2 and CH_2_-4/CH(OH)-6. The presence of the α,β-unsaturated ketone moiety constructed by the remaining three non-protonated sp^2^ carbons C-4 (δ_C_ 197.6), C-3′ (δ_C_ 147.7), and C-6′ (δ_C_ 187.7) was deduced by their characteristic chemical shifts and HMBC correlations (Figure 2) from both H_2_-5 and H-6 to C-4, C-3′, and C-6′. The connection between C-3 and C-3′ was revealed by the HMBC correlation from H_3_-7 to C-3′. The connections of C-6′/C-6 and C-3′/C-4 to construct a five-membered carbon ring were deduced by the long-range coupling (Figure 2) between H-3 and H-6 as well as the HMBC correlations from H_2_-5 to C-3′. The above data accounted for three out of four double-bond equivalent, indicating the presence of another ring in the molecule. According to the molecular formula, a sulfur atom was present in the structure of **1**. Considering the above, a linkage between C-2 and C-6′ through a sulfur atom was proposed, as also suggested by the chemical shifts of CH_2_-2 (δ_C/H_ 49.0/4.02, 3.39) and C-6′ (δ_C_ 187.7). The planar structure of **2** was assigned the same as **1** by their same 2D NMR data (Figure 2). Because there are only two stereogenic centers in the structure, **2** was thus assigned to be the C-3 or C-6 epimer of **1**. The determination of the relative configurations of **1** and **2** using NOESY spectra was failed for the far spatial distance between the two stereogenic centers, and the ^13^C NMR calculation method would not work either due to the nearly identical ^13^C NMR data between **1** and **2**. In order to determine the absolute configurations of **1** and **2**, quantum ECD calculations (Kong et al., 2019) of the four possible stereoisomers **a**, *ent*-**a**, **b**, and *ent*-**b** were thus performed (Figure 3 and Supplementary Figure 1), and the calculated UV and ECD curves were compared with the experimental ones of **1** and **2**. The results showed that the experimental UV curves of **1** and **2** were in good agreement with those of the calculated (Supplementary Figure 2), which further confirmed the planar structure of **1** and **2**. Furthermore, the ECD curves of **1** and **2** matched well with the calculated ECD curves of **a** and **b** (Figure 3), respectively, thus assigning the absolute configurations of **1** and **2** as (3*R*,6*R*)- and (3*R*,6*S*)-, respectively. Molecular orbital (MO) analysis (Supplementary Figure 3) revealed that the strong Cotton effects (CEs) around 300 and 325 nm in **1** and **2** were resulted from π→π^∗^ (MO45 → MO46) and n →π^∗^ (MO44 → MO46) transitions in the α,β-unsaturated ketone, respectively. It has been reported (Gawronski et al., 1996) that the signs of the CEs related to the π→π^∗^ and n →π^∗^ transitions of the α,β-unsaturated ketone in a five-membered ring correlated with the configuration of the chiral carbon at the γ position, i.e., C-6 in **1** and **2**. Thus, the nearly reverse CEs around 300 and 325 nm between **1** and **2** also confirmed their different configurations at C-6. Structurally, compounds **1** and **2** represented a new family of sulfur containing natural products with a 3-methyl-4*H*-cyclopenta[b]thiophen-4-one skeleton.

**FIGURE 4:**
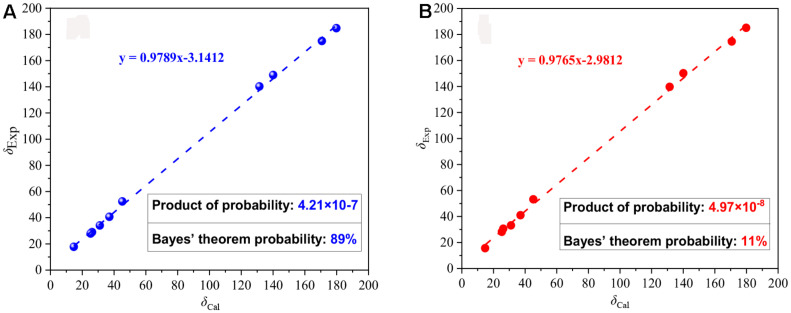
Linear correlations and DP4 probabilities between the experimental and calculated ^13^C NMR chemical shifts for **3 (A)** and 4-*epi*-**3 (B)**.

**FIGURE 5:**
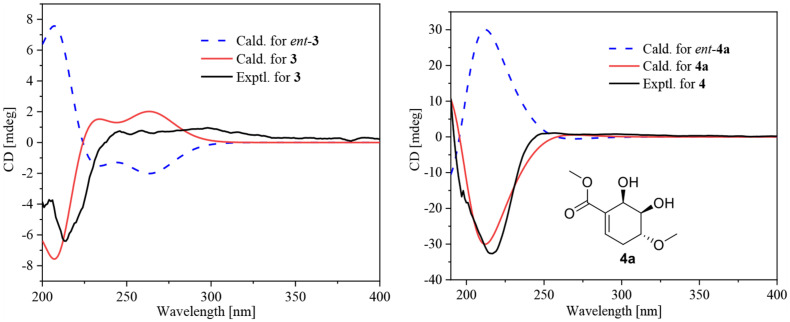
Experimental ECD curve for **3** and **4** and calculated ECD curves for **3**, *ent*-**3**, **4a**, and *ent*-**4a**.

Compound **3** was obtained as a yellow powder, and its molecular formula was determined as C_10_H_14_O_4_ according to the HRESIMS data, with four degrees of unsaturation. The ^13^C NMR and DEPT spectra displayed signals for two carboxylic carbonyls, one tri-substituted double bond, three sp^3^ methylenes, two sp^3^ methines, and one methyl. In the COSY spectrum (Figure 2), correlations of H_3_-10/H-8/H-4/H_2_-5/H_2_-6 and H-8/H_2_-3/H-2 were observed. In the HMBC spectrum (Figure 2), H_3_-10 correlated with the C-9 carboxylic carbonyl, and H_2_-6 and H-2 correlated with both C-1 and C-9 carboxylic carbonyl. The above data led to the determination of the planar structure of **3**. In order to determine the relative configuration of **3**, the ^13^C NMR calculations (Kong et al., 2019) of **3** and 4-*epi*-**3** at the B3LYP/6-311 + + G(2d,p) level were performed (Figure 4 and Supplementary Figures 4, 5). As shown in Figure 4, the calculated NMR chemical shifts of **3** (Figure 4A) coincided better with the experimental data compared to those of 4-*epi*-**3** (Figure 4B). The DP4 probability analysis (Lv et al., 2019) also showed that **3** had 89% probability while only 11% for 4-*epi*-**3** (Figure 4 and Supplementary Table 1). These data led to the assignment of the relative configuration of **3**. Based on the above assignment, the ECD curves of **3** and *ent*-**3** were calculated (Figure 5). The calculated ECD curve for **3** matched well with the experimental one, thus assigning the absolute configuration as (4*S*,8*R*)-.

**FIGURE 6:**
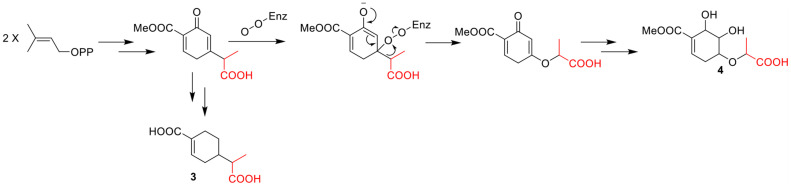
Hypothetical biogenetic pathway of compound **3** and **4**.

The molecular formula of compound **4** was established as C_11_H_16_O_7_ by HRESIMS. The double-bond equivalent of **4** was calculated to be four. The HSQC spectrum indicated the presence of four oxymethines, one sp3 methylene, and two methyl with one methoxy, one carboxylic acid and a α,β-unsaturated carboxylic acid or ester group. The presence of two carbonyls and one tri-substituted double bond (Supplementary Table 1) indicated that **4** has a ring in its structure. Analysis of the COSY data (Figure 2) suggested the presence of the substructures CH-2/CH_2_-3/CH-4/CH-5/CH-6 and CH_3_-10/CH-8. HMBC correlations from H-2 and H-6 to C-1 and C-7 carbonyl led to the construction of the skeleton of **4**. HMBC correlations from CH_3_-10 to the carboxylic carbonyl C-9 and from H-8 to the oxymethine C-4 led to the assignment of the full structure of **4**. The large *J* value (7.1 Hz) (Table 2) between H-4 and H-5 indicated their *trans* relationship, while the small *J* value (4.7 Hz) between H-5 and H-6 suggested their *cis* orientation. In order to assign the absolute configuration of **4**, the ECD curves of the simplified structure **4a** of **4** was calculated and the resulted ECD curve showed good agreement with the experimental ECD spectrum (Figure 5), thus assigning the absolute configurations of C-4, C-5, and C-6 as *R*, *R*, and *R*, respectively. The skeletons of **3** and **4** are very similar with the only difference being the presence of an oxygen atom between C-4 and C-8 in **4**, which suggested that the biosynthetic pathways of **3** and **4** are closely related. A biosynthetic pathway including a Baeyer-Villiger like oxidation key step to afford **4** was thus proposed (Figure 6). Based on the biosynthetic consideration, the absolute configuration of C-8 of **4** was tentatively assigned to be *S* according to the assigned C-8 configuration of **3**.

### Bioactivity Assay

**FIGURE 7:**
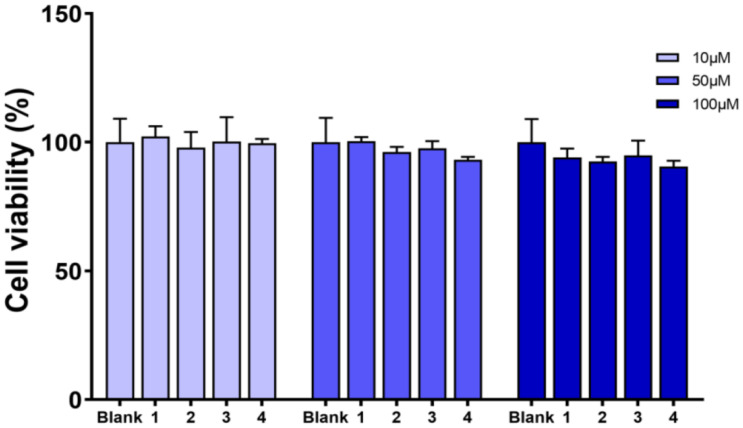
Viability effects of compounds **1–4** against RAW264.7 cells.

**FIGURE 8:**
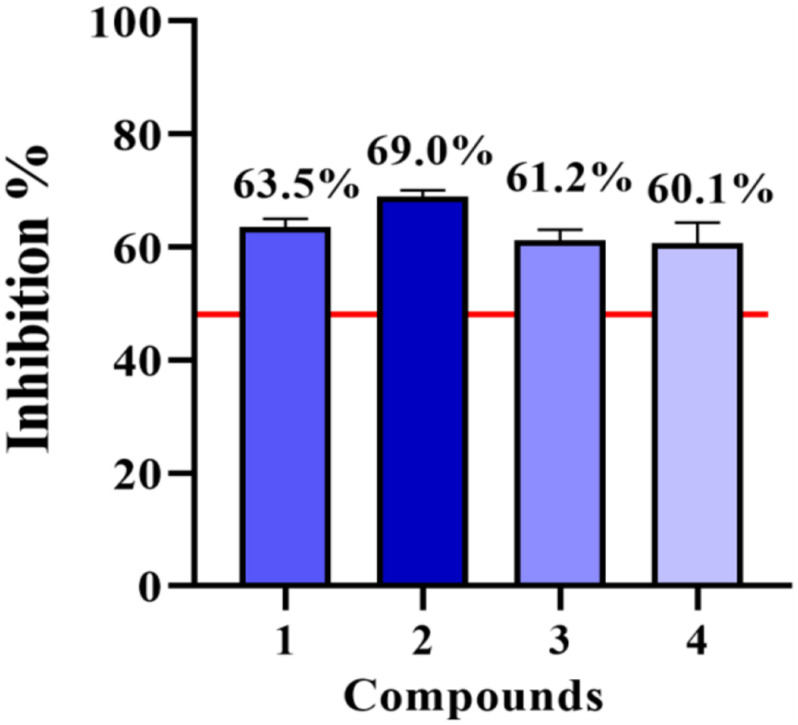
NO inhibitory activities of compounds **1–4** at 80 μM.

Compounds **1**–**4** were evaluated for cell viability using MTT assay. As shown in Figure 7, all the compounds have no obvious effect on the cell viability of RAW264.7 cells at three different concentrations (10, 50, and 100 μM) (Figure 7). Based on this, the suppressive effects of compounds **1**–**4** on the over-production of NO in cells were tested (Cao et al., 2021) at the concentration of 80 μM. The result showed that all the tested compounds were active (Figure 8). The IC_50_ values of compounds **1**–**4** against the over-production of NO in cells were finally determined to be 38.0, 19.8, 46.3, and 56.6 μM, respectively (positive control dexamethasone, IC_50_: 7.5 μM). Besides, the antibacterial activity of **1**–**4** against *Staphylococcus aureus* were also evaluated (Pierce et al., 2008), and all of the compounds were inactive.

## Conclusion

In summary, from the fungus *Aspergillus* sp. CYH26, two new thio compounds (**1** and **2**) and two new monoterpenes (**3** and **4**) were isolated and identified. Compounds **1** and **2** are identified as rare sulfur containing natural products with a 3-methyl-4*H*-cyclopenta[b]thiophen-4-one skeleton, which are first encountered in nature. Compounds **1**–**4** could suppress the over-production of NO without affecting the cell viability. These results further demonstrated that fungi is an abundant source of new bioactive products with medicinal use.

## Data Availability Statement

The datasets presented in this study can be found in online repositories. The names of the repository/repositories and accession number(s) can be found in the article/Supplementary Material.

## Author Contributions

GP conceived and designed the experiments, and was involved in isolation of compounds. YL, XC, DT, WH, ZW, and YZ contributed to isolation of compounds. SR, GH, and YX performed genetic manipulation, strain fermentation, and extraction. MG contributed to the collection of the physicochemical data of compounds. NX contributed to bioactivity assay and revised the manuscript. FK supervised the work and prepared the manuscript. All authors contributed to the article and approved the submitted version.

## Conflict of Interest

The authors declare that the research was conducted in the absence of any commercial or financial relationships that could be construed as a potential conflict of interest.
