# Supplementary material for: New Thio-Compounds and Monoterpenes With Anti-inflammatory Activities From the Fungus Aspergillus sp. CYH26
Source: Front Microbiol. 2021 Mar 25;12:668938. doi: 10.3389/fmicb.2021.668938 (PMC8025228; doi:10.3389/fmicb.2021.668938)

Supplementary Material

New Thio Compounds and Monoterpenes with anti-Inflammatory Activities from the Fungus *Aspergillus* sp. CYH26

Guojun Pan, [1] Yanling Li, [1] Xinyu Che, [2] Dan Tian, [2] Wenjie Han, [1] Zimin Wang, [1] Yanfen Zhao, [1] Shuang Ren, [1] Yiru Xu, [1] Gangping Hao, [1] Mengfei Guo, [3] Na Xiao, [2, 4*] Fandong Kong [3*]

[1] *College of Life Sciences, Shandong First Medical University & Shandong Academy of Medical Sciences, Tai'an, Shandong 271000, China.*
[2] *State Key Laboratory of Crop Biology, college of Agromomy, Shandong Agriculture University, Tai'an, Shandong 271018, China.*
[3] *Key Laboratory of Chemistry and Engineering of Forest Products, State Ethnic Affairs Commission, Guangxi Key Laboratory of Chemistry and Engineering of Forest Products, Guangxi Collaborative Innovation Center for Chemistry and Engineering of Forest Products, School of Chemistry and Chemical Engineering, Guangxi University for Nationalities, Nanning 530006, China.*
[4] *State Key Laboratory of Natural Medicines, China Pharmaceutical University, Nanjing 210009, China.*

List of Supporting Information

The ITS gene sequences of *Aspergillus* sp. CYH26……S3
Theory and Calculation Details……S3
Figure S1. DFT-optimized low-energy conformers of **a** and **b**……S4
Figure S2. The experimental and calculated UV spectra for compound **1**……S4
Figure S3. MOs 44, 45, and 46 involved in important transitions in the ECD spectrum of the lowest-energy conformer of **a**……S4.
Figure S4. DFT-optimized low-energy conformers of **3.** ……S5.
Figure S5. DFT-optimized low-energy conformers of 4-***epi*-3**……S5.
Figure S6. DFT-optimized low-energy conformers of the simplified structure **4a** of **4**. ……S5.
Table S1. The calculated ^{13}C NMR data for **3** and 4-*epi*-**3**……S6.
Figure S7. ^{1}H-NMR spectrum of **1** in CD_3OD……S7.
Figure S8. ^{13}C-NMR spectrum of **1** in CD_3OD……S7.
Figure S9. HSQC spectrum of **1** in CD_3OD……S8.
Figure S10. COSY spectrum of **1** in CD_3OD……S8.
Figure S11. HMBC spectrum of **1** in CD_3OD……S9.
Figure S12. NOESY spectrum of **1** in CD_3OD……S9.

Figure S13. HRESIMS spectrum of **1** in CD_3OD..S10.
Figure S14. ^{1}H-NMR spectrum of **2** in CD_3OD..S11.
Figure S15. ^{13}C-NMR spectrum of **2** in CD_3OD..S11.
Figure S16. HSQC spectrum of **2** in CD_3OD..S12.
Figure S17. COSY spectrum of **2** in CD_3OD..S12.
Figure S18. HMBC spectrum of **2** in CD_3OD..S13.
Figure S19. NOESY spectrum of **2** in CD_3OD..S13.
Figure S20. HRESIMS spectrum of **2** in CD_3OD..S14.
Figure S21. ^{1}H-NMR spectrum of **3** in CD_3OD..S15.
Figure S22. ^{13}C-NMR spectrum of **3** in CD_3OD..S15.
Figure S23. HSQC spectrum of **3** in CD_3OD..S16.
Figure S24. COSY spectrum of **3** in CD_3OD..S16.
Figure S25. HMBC spectrum of **3** in CD_3OD..S17.
Figure S26. NOESY spectrum of **3** in CD_3OD..S17.
Figure S27. HRESIMS spectrum of **3** in CD_3OD..S18.
Figure S28. ^{1}H-NMR spectrum of **4** in CD_3OD..S19.
Figure S29. ^{13}C-NMR spectrum of **4** in CD_3OD..S19.
Figure S30. HSQC spectrum of **4** in CD_3OD..S20.
Figure S31. COSY spectrum of **4** in CD_3OD..S20.
Figure S32. HMBC spectrum of **4** in CD_3OD..S21.
Figure S33. NOESY spectrum of **4** in CD_3OD..S21.
Figure S34. HRESIMS spectrum of **4** in CD_3OD..S22.

The ITS sequence of *Aspergillus* sp. CYH26

CACCTGGAAAGAATGGTTGGAAAACGTCGGCAGGCGCCGGCCAATCCTACAGAGCAT
GTGACAAAGCCCCATACGCTCGAGGATCGGACGCGGTGCCGCCGCTGCCTTTCGGGCC
CGTCCCCCCGGAGAGGGGGACGGCGACCCAACACACAAGCCGGGCTTGAGGGCAGC
AATGACGCTCGGACAGGCATGCCCCCCGGAATACCAGGGGGCGCAATGTGCGTTCAAA
GACTCGATGATTCACTGAATTCTGCAATTCACATTAGTTATCGCATTTCGCTGCGTTCTT
CATCGATGCCGGAACCAAGAGATCCATTGTTGAAAGTTTTAACTGATTGCATTCAATCA
ACTCAGACTGCACGCTTTCAGACAGTGTTCGTGTTGGGGTCTCCGGCGGGCACGGGCC
CGGGGGGCAGAGGCGCCCCCCCGGCGGCCGACAAGCGGCGGGCCCGCCGAAGCAAC
AGGGTACAATAGACACGGATGGGAGGTTGGGCCCAAAGGACCCGCACTCGGTAATGAT
CCTTCCGCAGGTTCACCTACGGAAGGG

ECD and ^{13}CNMR calculation section

Theory and Calculation Details.

The calculations were performed by using the density functional theory (DFT) as carried out in the Gaussian 16.[S1]The preliminary conformational distributions search was performed using Frog2 online version[S2]. Further geometrical optimization were performed at the B3LYP/6-31G(d) level. Solvent effects of methanol solution were evaluated at the same DFT level by using the SCRF/PCM method.[S3] TDDFT[S4] at B3LYP/6-31G(d) was employed to calculate the electronic excitation energies and rotational strengths in methanol. The stable conformations obtained at the B3LYP/6-31G(d) level were further used in magnetic shielding constants at the B3LYP/6-311++G(2d,p) level.

(S1) Gaussian 16, Revision C.01, M. J. Frisch, G. W. Trucks, H. B. Schlegel, G. E. Scuseria, M. A. Robb, J. R. Cheeseman, G. Scalmani, V. Barone, G. A. Petersson, H. Nakatsuji, X. Li, M. Caricato, A. V. Marenich, J. Bloino, B. G. Janesko, R. Gomperts, B. Mennucci, H. P. Hratchian, J. V. Ortiz, A. F. Izmaylov, J. L. Sonnenberg, D. Williams-Young, F. Ding, F. Lipparini, F. Egidi, J. Goings, B. Peng, A. Petrone, T. Henderson, D. Ranasinghe, V. G. Zakrzewski, J. Gao, N. Rega, G. Zheng, W. Liang, M. Hada, M. Ehara, K. Toyota, R. Fukuda, J. Hasegawa, M. Ishida, T. Nakajima, Y. Honda, O. Kitao, H. Nakai, T. Vreven, K. Throssell, J. A. Montgomery, Jr., J. E. Peralta, F. Ogliaro, M. J. Bearpark, J. J. Heyd, E. N. Brothers, K. N. Kudin, V. N. Staroverov, T. A. Keith, R. Kobayashi, J. Normand, K. Raghavachari, A. P. Rendell, J. C. Burant, S. S. Iyengar, J. Tomasi, M. Cossi, J. M. Millam, M. Klene, C. Adamo, R. Cammi, J. W. Ochterski, R. L. Martin, K. Morokuma, O. Farkas, J. B. Foresman, and D. J. Fox, Gaussian, Inc., Wallingford CT, 2019.

(S2) Miteva, M. A.; Guyon, F.; and Tuffery, P.; *Nucleic Acids Res.*, **2010**, *38*, W622–W627.

(S3) Sai, C.; Li, D.; Xue, C.; Wang, K.; Hu, P.; Pei, Y.; Bai, J.; Jing Y.; Li, Z.; Hua H.

Org. Lett. **2015**, *17*, 4102-5.

(S4) (a) Miertus, S.; Tomasi, *J. Chem. Phys.* **1982**, *65*, 239–245. (b) Tomasi, J.; Persico, M. *Chem.Rev.* **1994**, *94*, 2027–2094. (c) Cammi, R.; Tomasi, J. J. *Comp.Chem.* **1995**, *16*, 1449–1458.

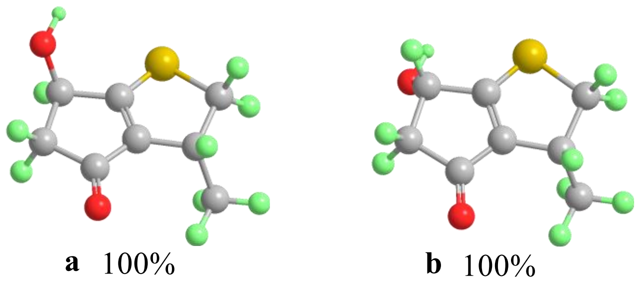

Figure S1. DFT-optimized low-energy conformer of **a** and **b**.

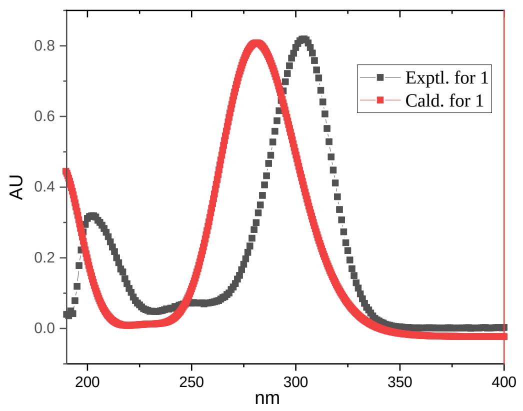

Figure S2. The experimental and calculated UV spectra for compound **1**.

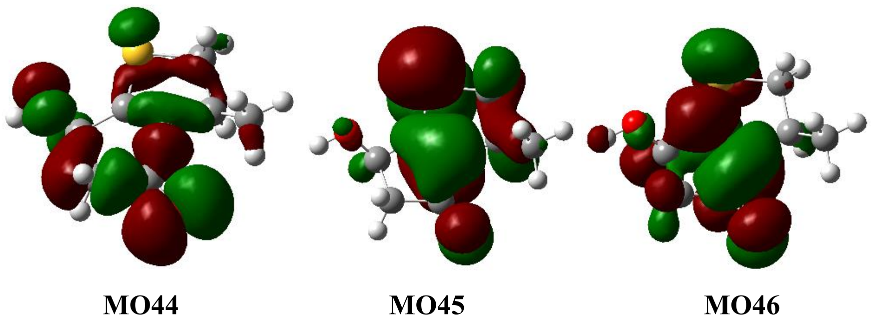

Figure S3. MOs 44, 45, and 46 involved in important transitions in the ECD spectrum of the lowest-energy conformer of **a**.

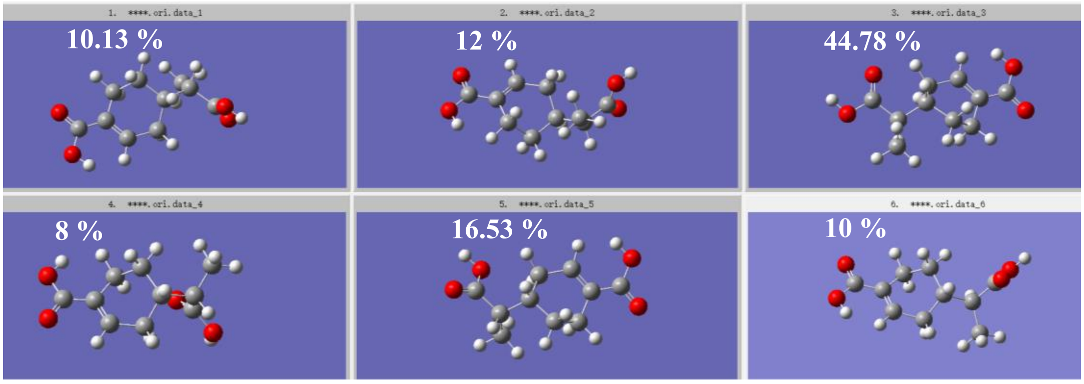

Figure S4. DFT-optimized low-energy conformers of **3.**

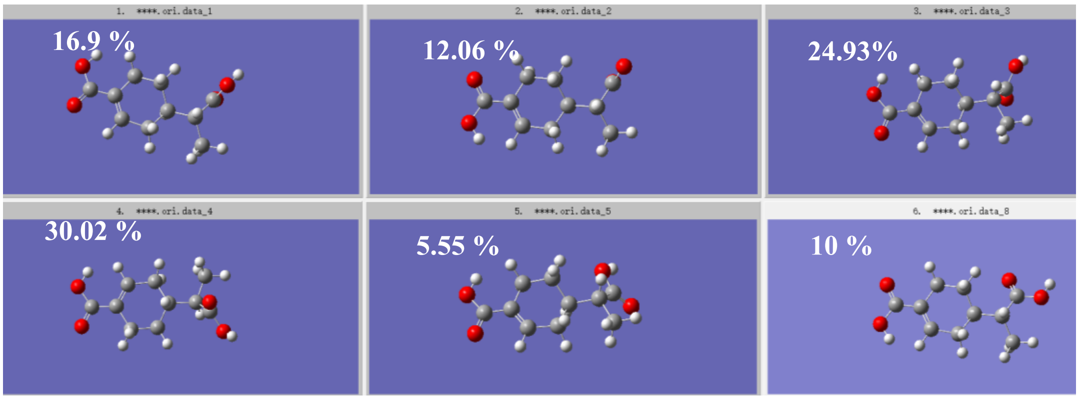

Figure S5. DFT-optimized low-energy conformers of 4-***epi*-3**.

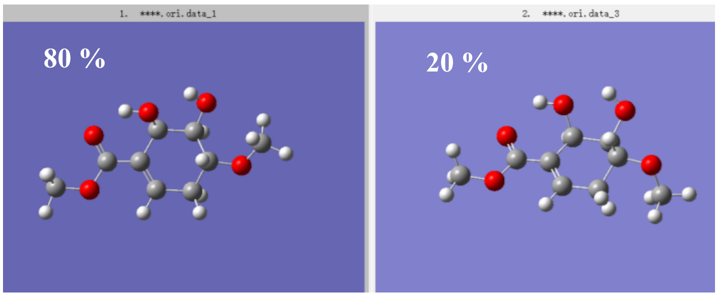

Figure S6. DFT-optimized low-energy conformers of the simplified structure **4a** of **4**.

Table S1. The calculated 13C NMR data for **3** and 4-*epi*-**3**

***δ*exp**	***δ*cal**		***δ*scal**		**corrected error**		**t distribution**		**probability**	
	**3**	**4-*epi*-3**	**3**	**4-*epi*-3**	**3**	**4-*epi*-3**	**3**	**4-*epi*-3**	**3**	**4-*epi*-3**
179.8	184.8	185.1	177.8	177.8	2.0	2.0	0.80	0.80	0.20	0.20
170.7	175	174.6	168.2	167.5	2.5	3.2	0.85	0.90	0.15	0.10
140.1	149	150.2	142.7	143.7	-2.6	-3.6	0.86	0.93	0.14	0.07
131.4	140.3	139.7	134.2	133.4	-2.8	-2.0	0.87	0.80	0.13	0.20
45.2	52.4	53.2	48.2	49.0	-3.0	-3.8	0.89	0.93	0.11	0.07
37.1	40.7	41	36.7	37.1	0.4	0.0	0.57	0.51	0.43	0.49
31.1	34.1	33.2	30.2	29.4	0.9	1.7	0.64	0.76	0.36	0.24
26.2	29	30.6	25.2	26.9	1.0	-0.7	0.66	0.62	0.34	0.38
25.3	27.9	28.2	24.2	24.6	1.1	0.7	0.68	0.62	0.32	0.38
14.7	17.8	15.6	14.3	12.3	0.4	2.4	0.57	0.84	0.43	0.16
**Product of probabilities**									4.21×10^{-7}	4.97×10^{-7}
**Bayes's theorem probability (%)**									**0.89**	**0.11**

Figure S7. ^{1}H-NMR spectrum of 1 in CD_3OD

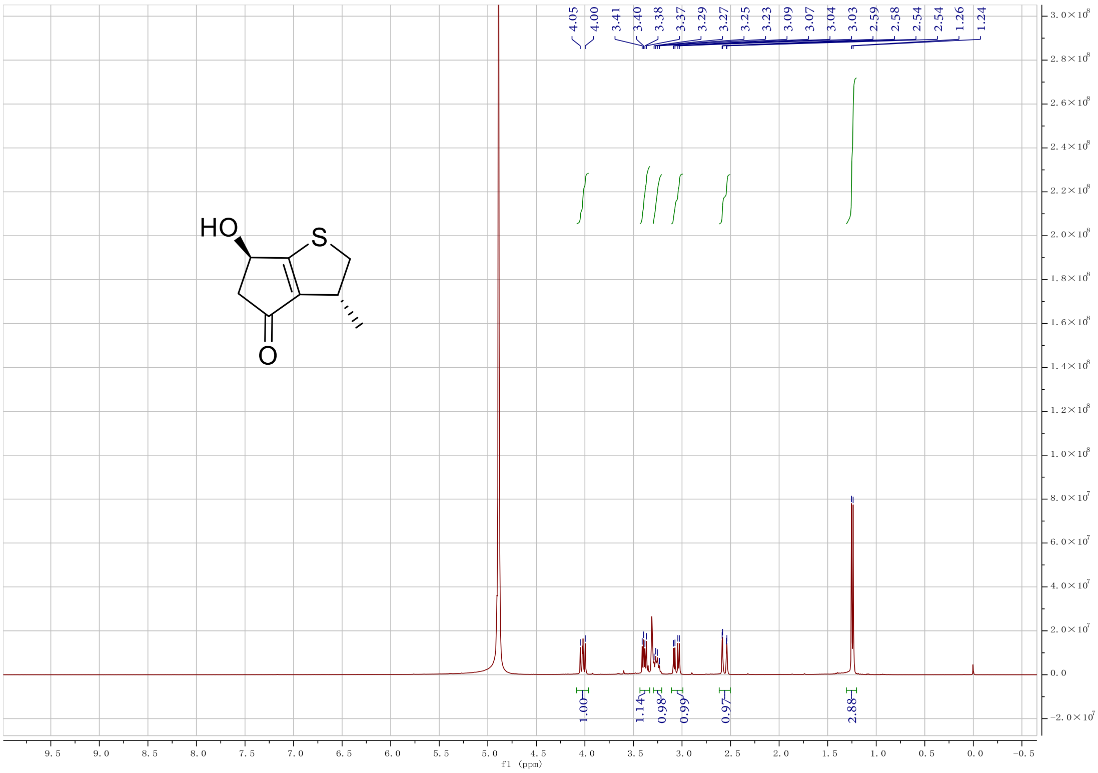

Figure S8. ^{13}C-NMR spectrum of 1 in CD_3OD

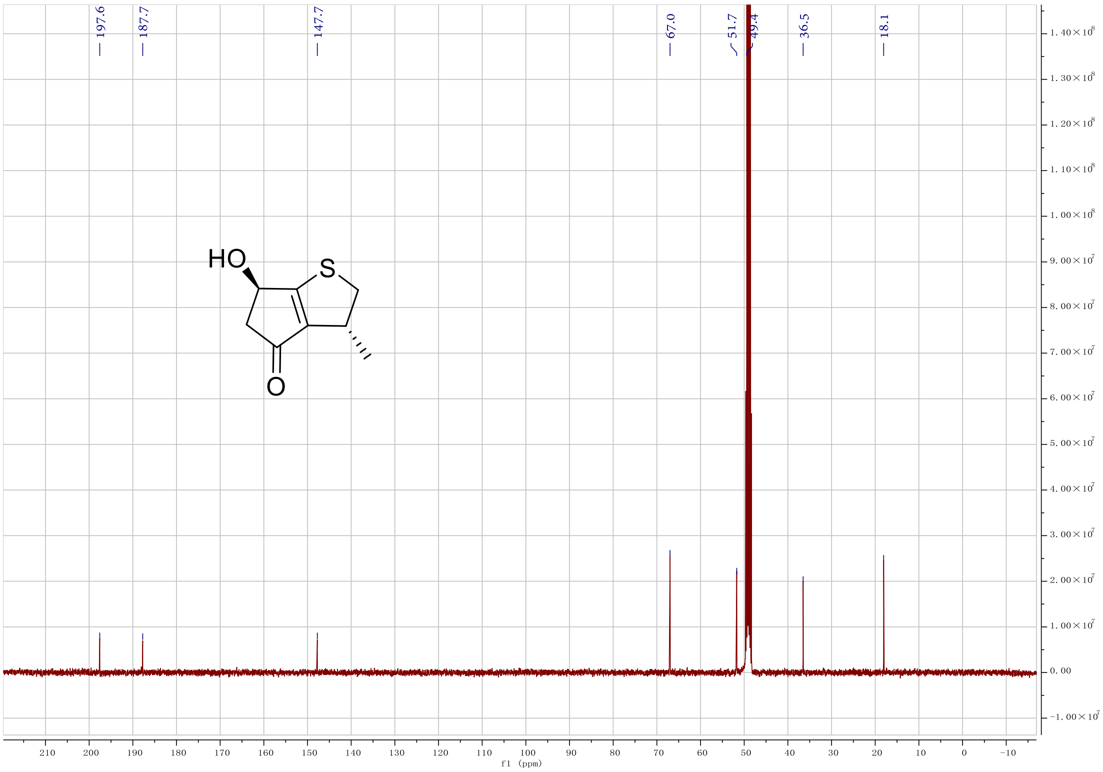

Figure S9. **HMQC spectrum of 1 in CD_3OD**

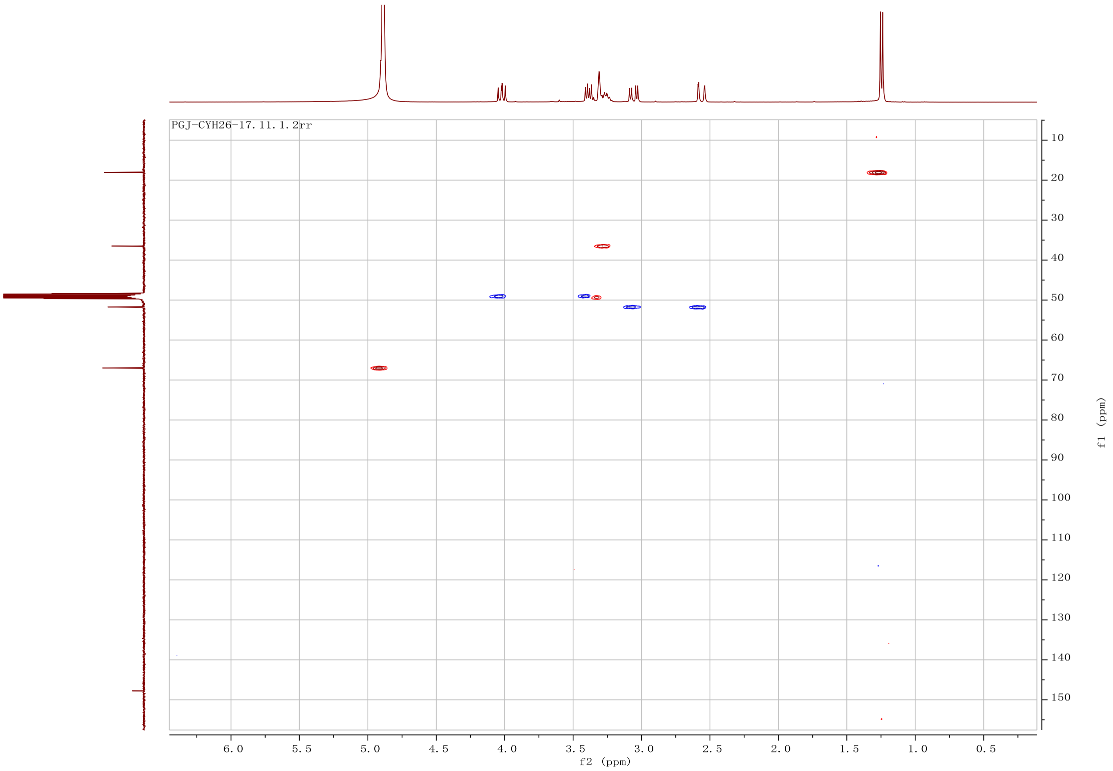

Figure S10. **^1H-^1H COSY spectrum of 1 in CD_3OD**

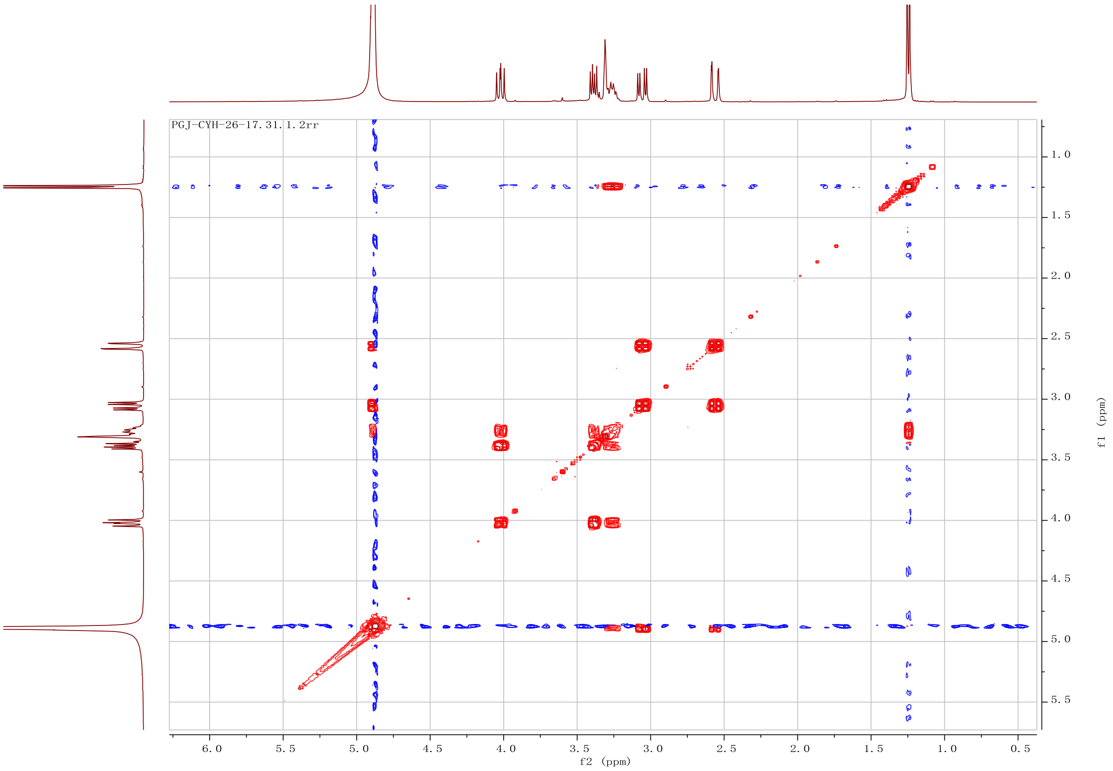

Figure S11. HMBC spectrum of 1 in CD_3OD

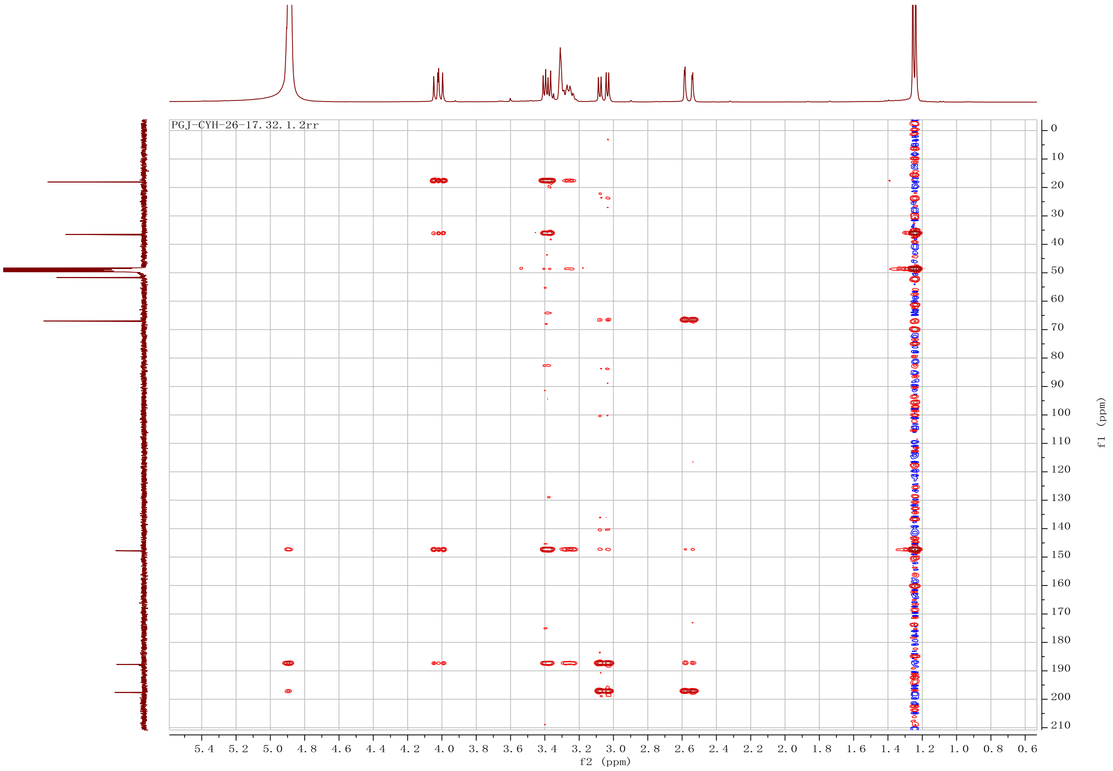

Figure S12. NOESY spectrum of 1 in CD_3OD

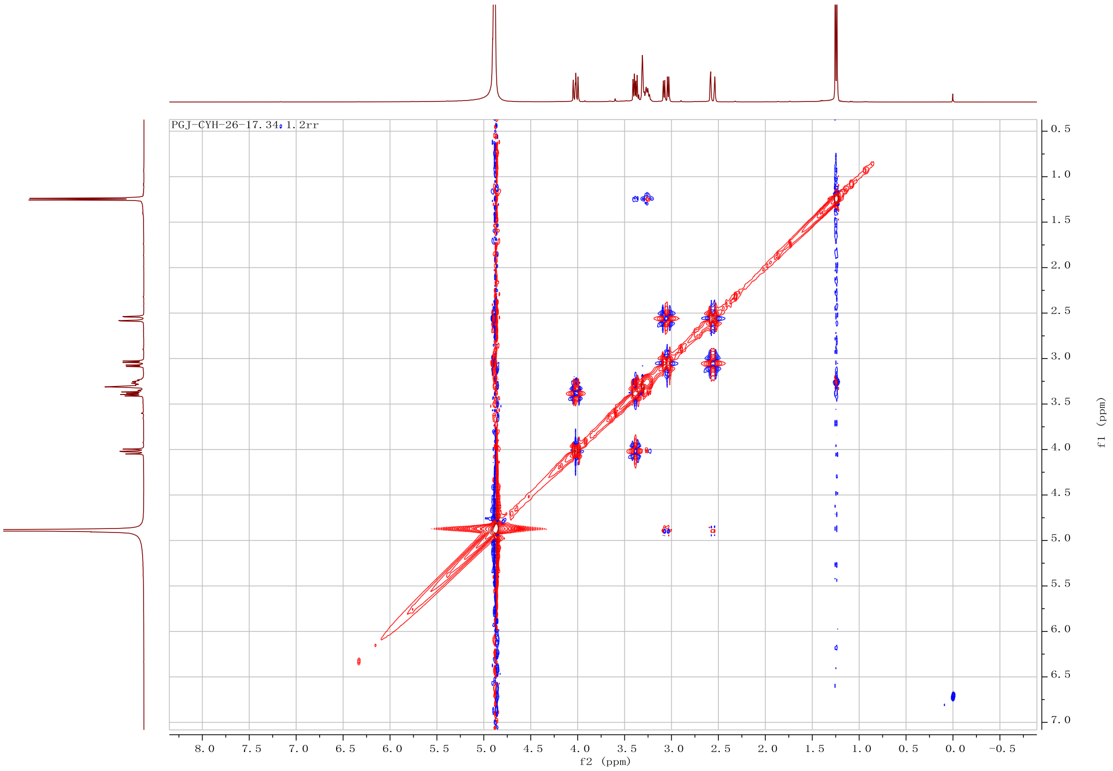

Figure S13. **HRESIMS spectrum of 1**

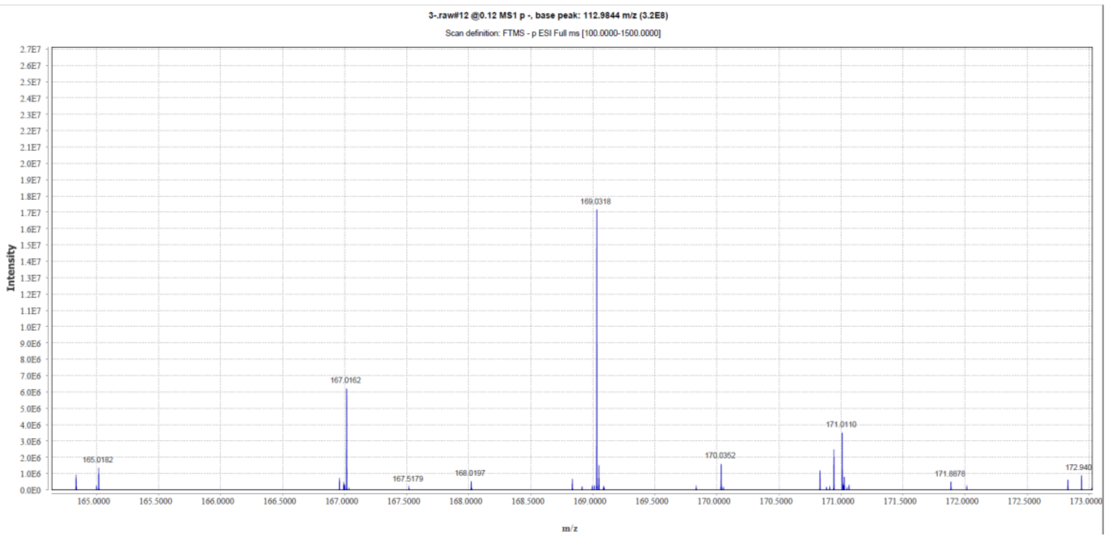

Figure S14. ^{1}H-NMR spectrum of 2 in CD_3OD

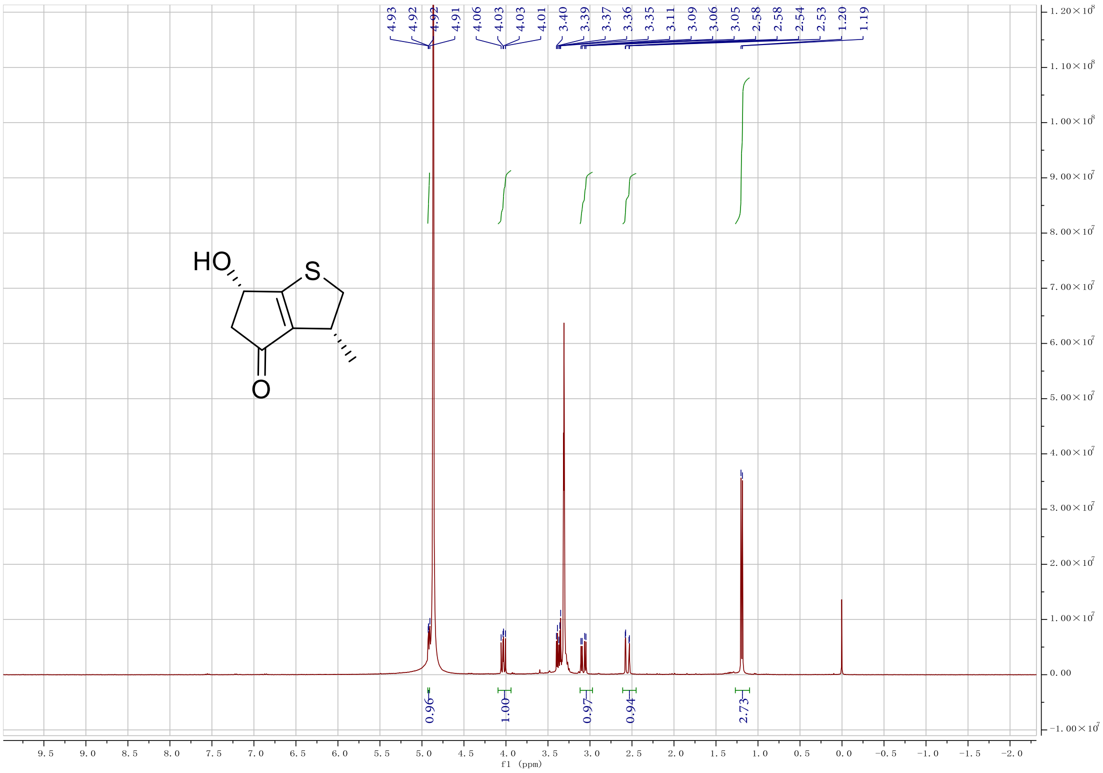

Figure S15. ^{13}C-NMR spectrum of 2 in CD_3OD

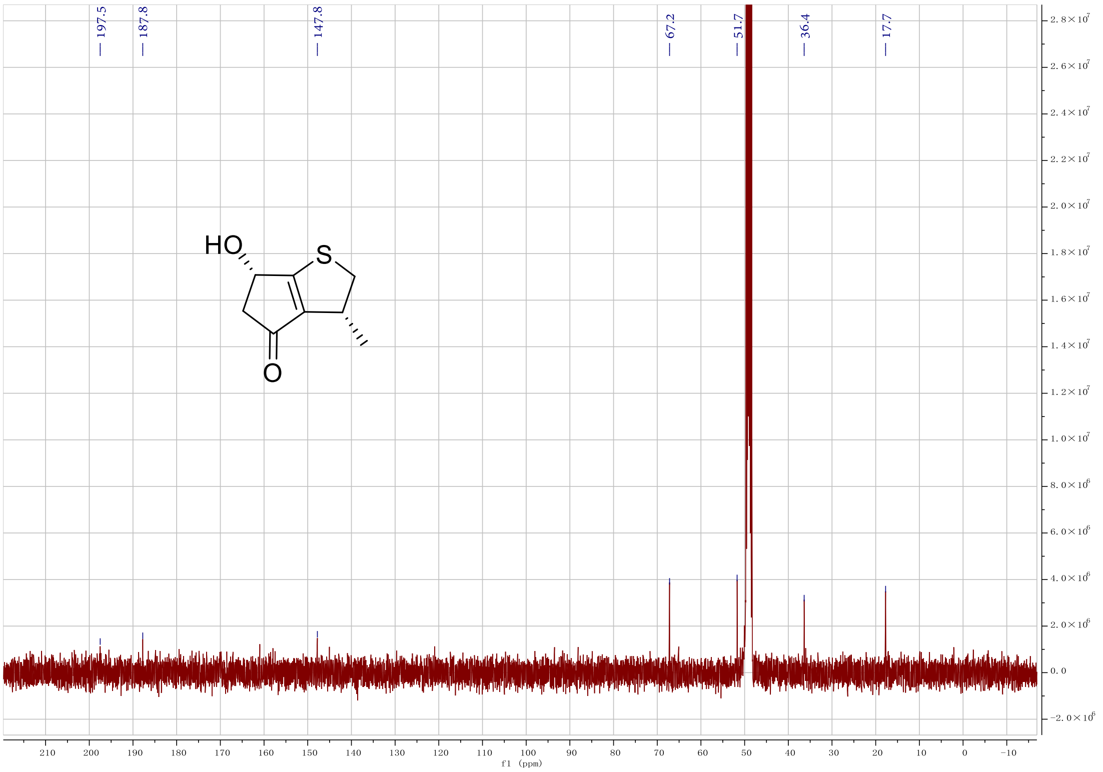

Figure S16. HMQC spectrum of 2 in CD_3OD

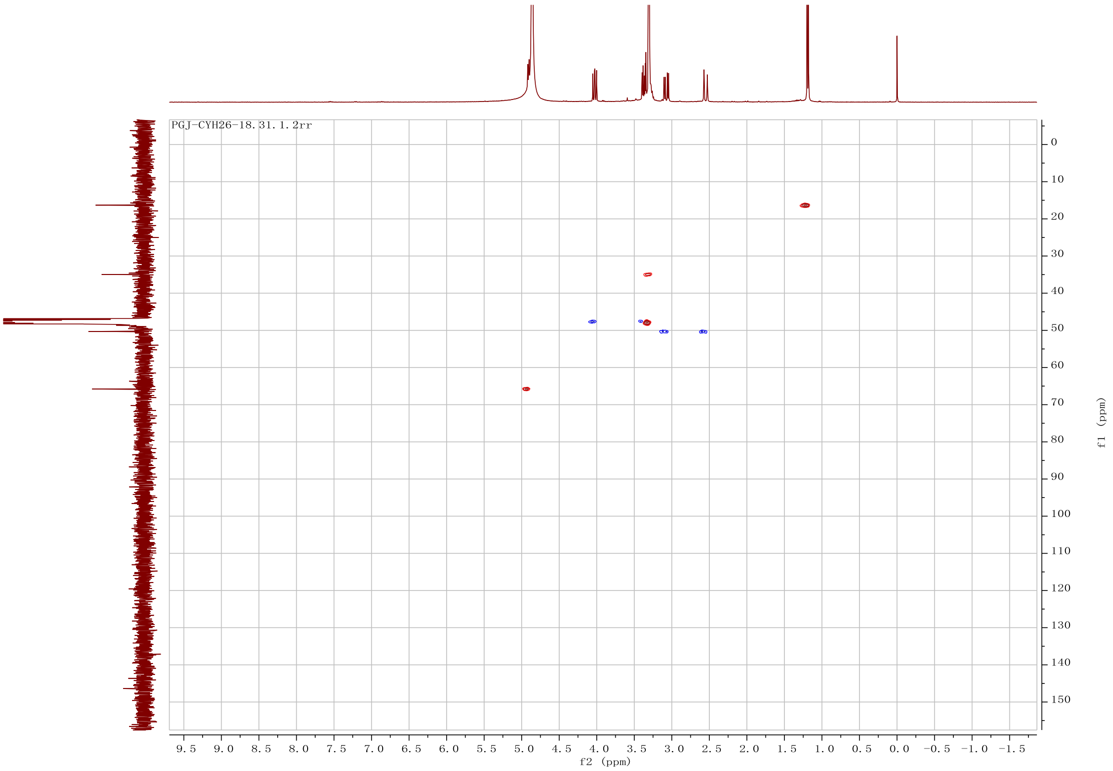

Figure S17. ^1H-^1H COSY spectrum of 2 in CD_3OD

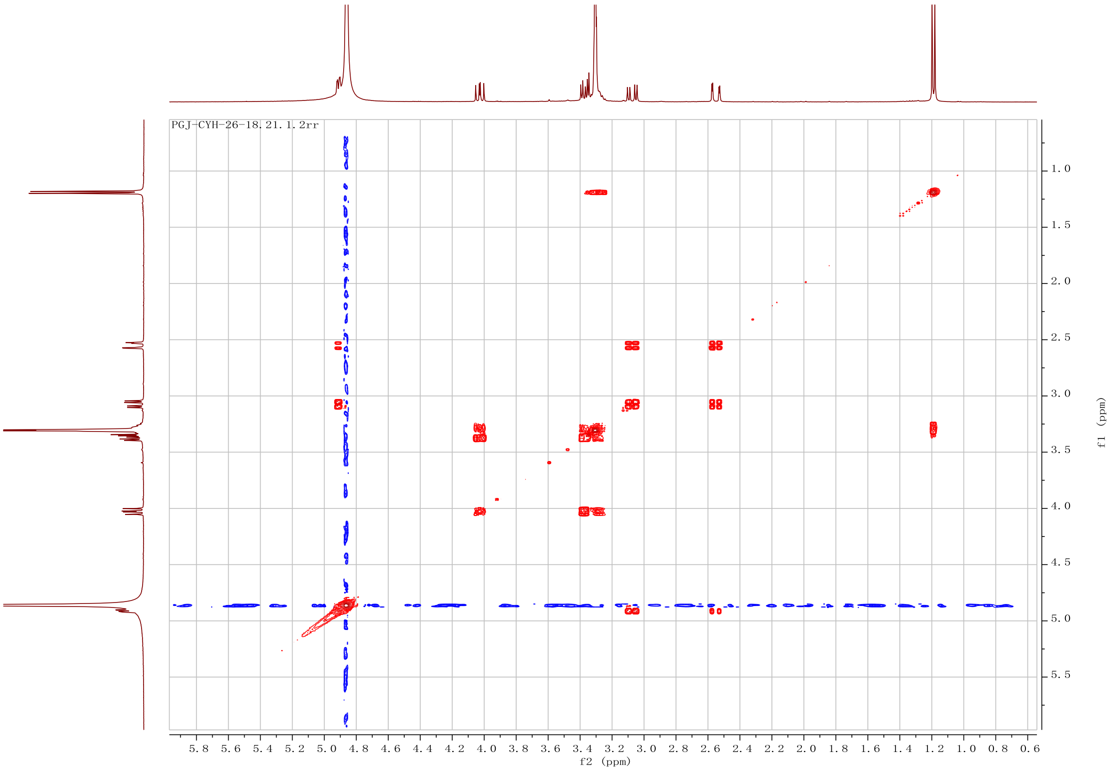

Figure S18. HMBC spectrum of 2 in CD_3OD

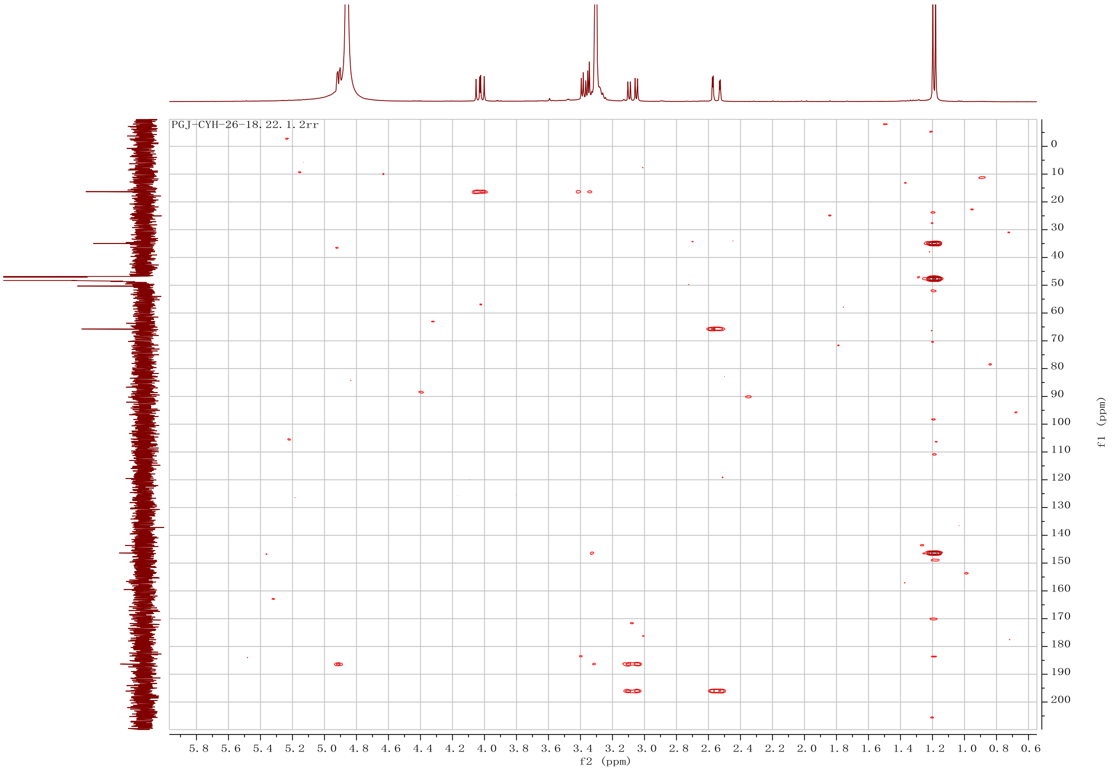

Figure S19. NOESY spectrum of 2 in CD_3OD

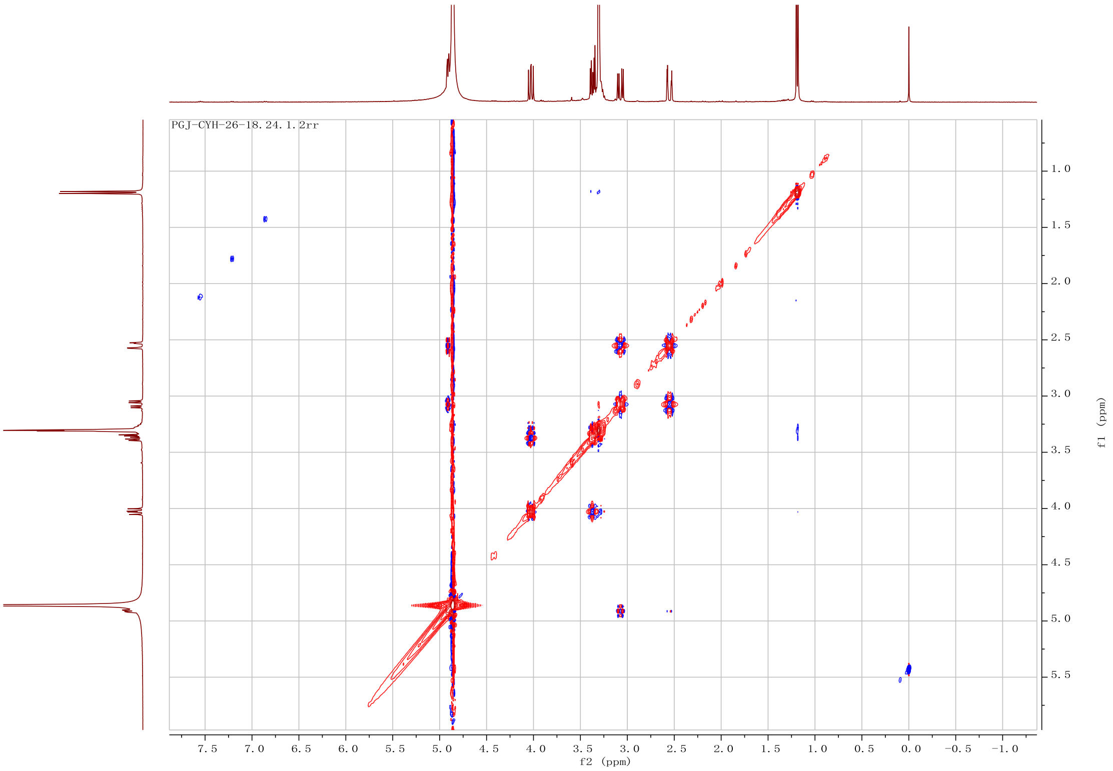

Figure S20. **HRESIMS spectrum of 2**

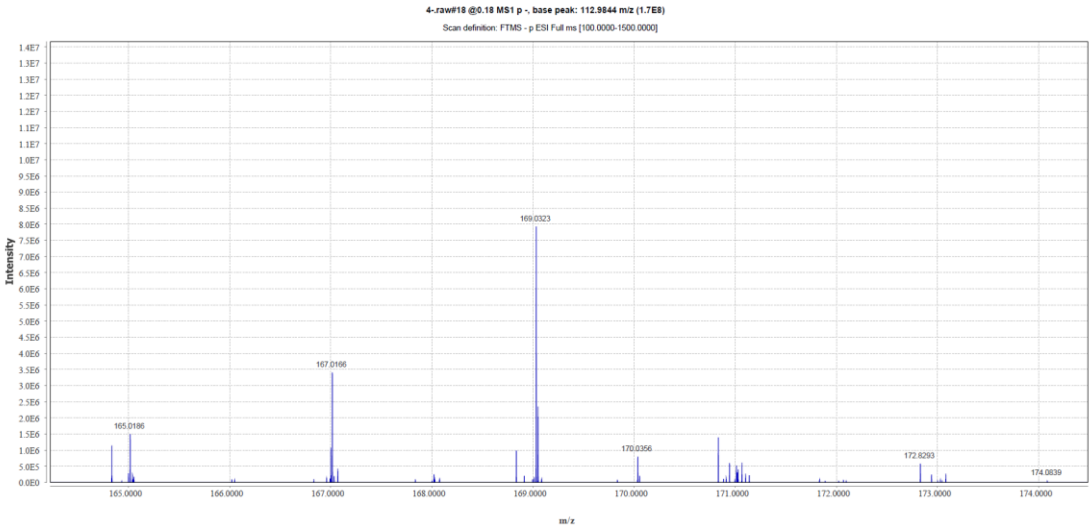

Figure S21. ^{1}H-NMR spectrum of 3 in CD_3OD

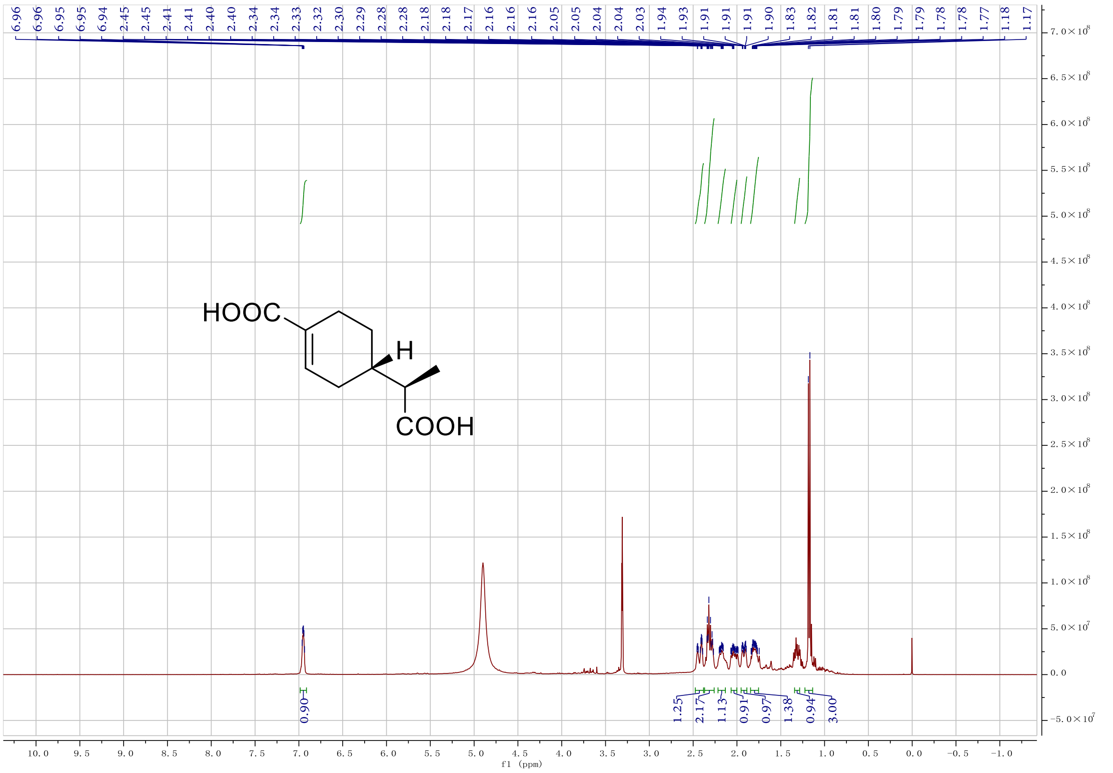

Figure S22. ^{13}C-NMR spectrum of 3 in CD_3OD

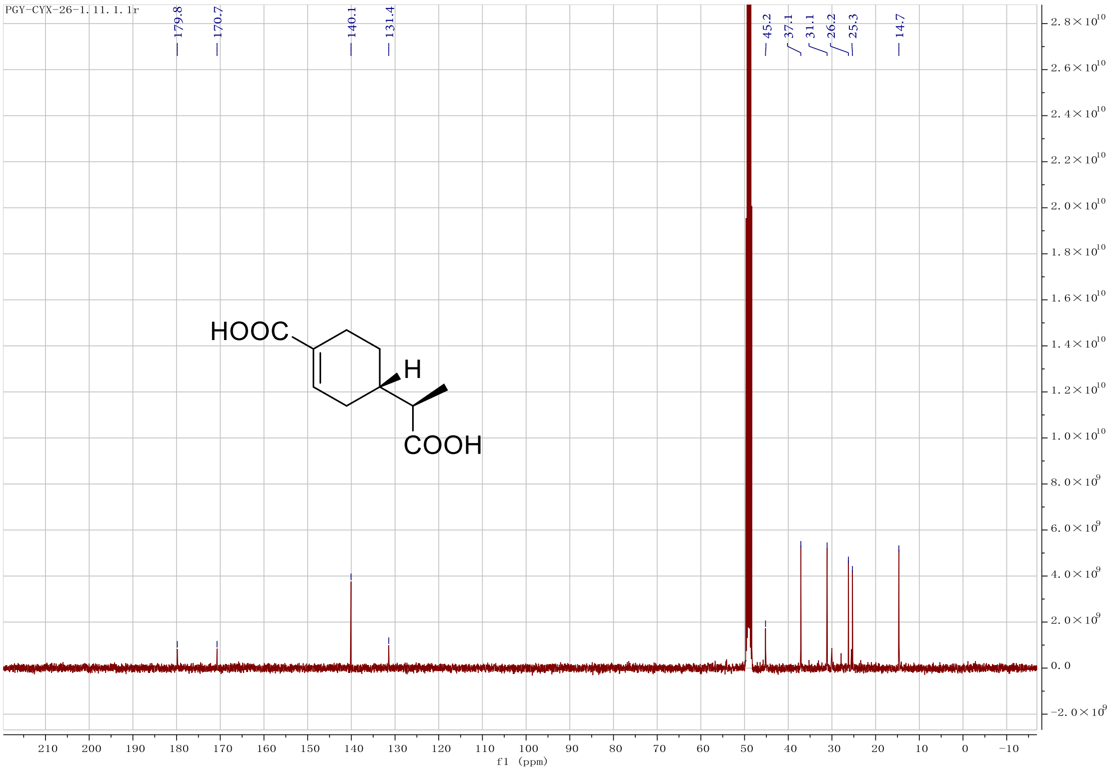

Figure S23. **HMQC spectrum of 3 in CD_3OD**

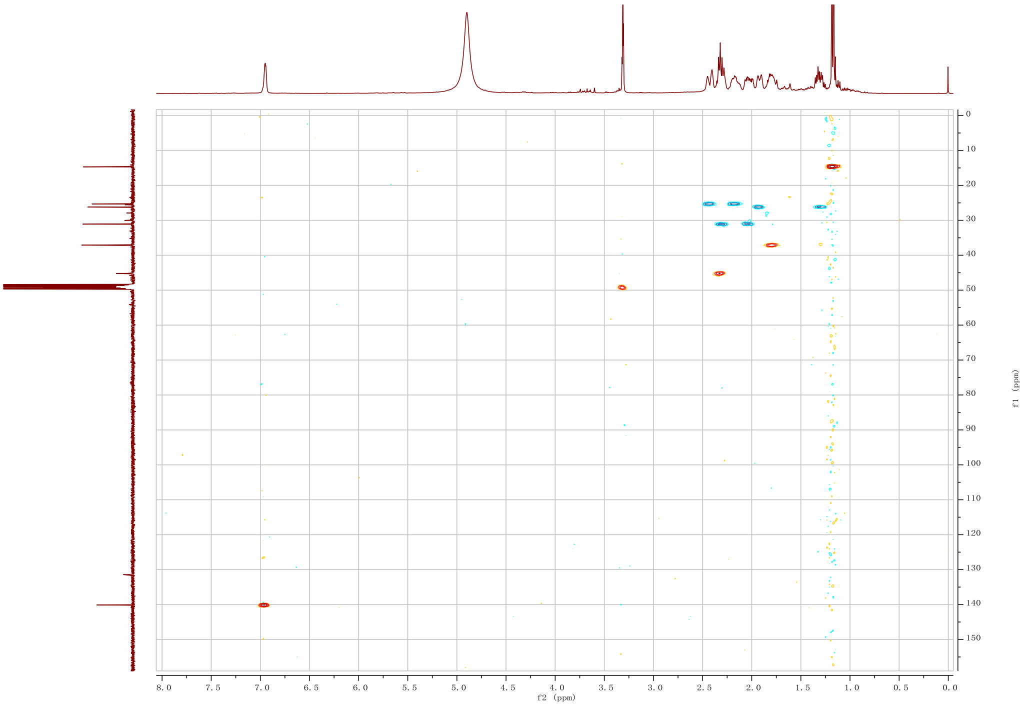

Figure S24. **^1H-^1H COSY spectrum of 3 in CD_3OD**

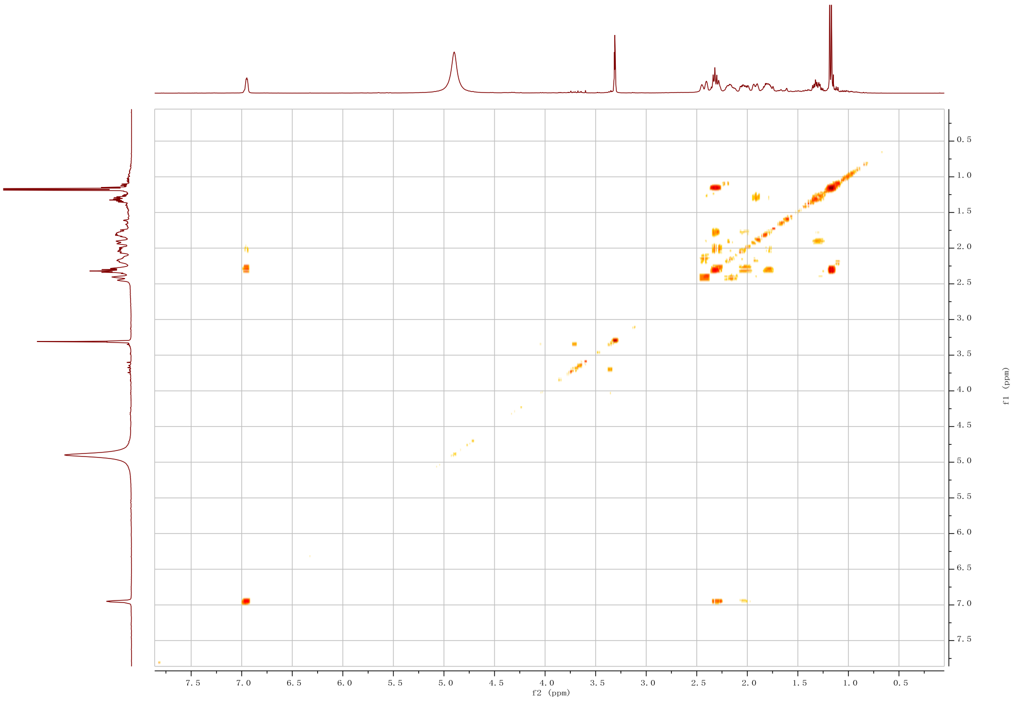

Figure S25. HMBC spectrum of 3 in CD_3OD

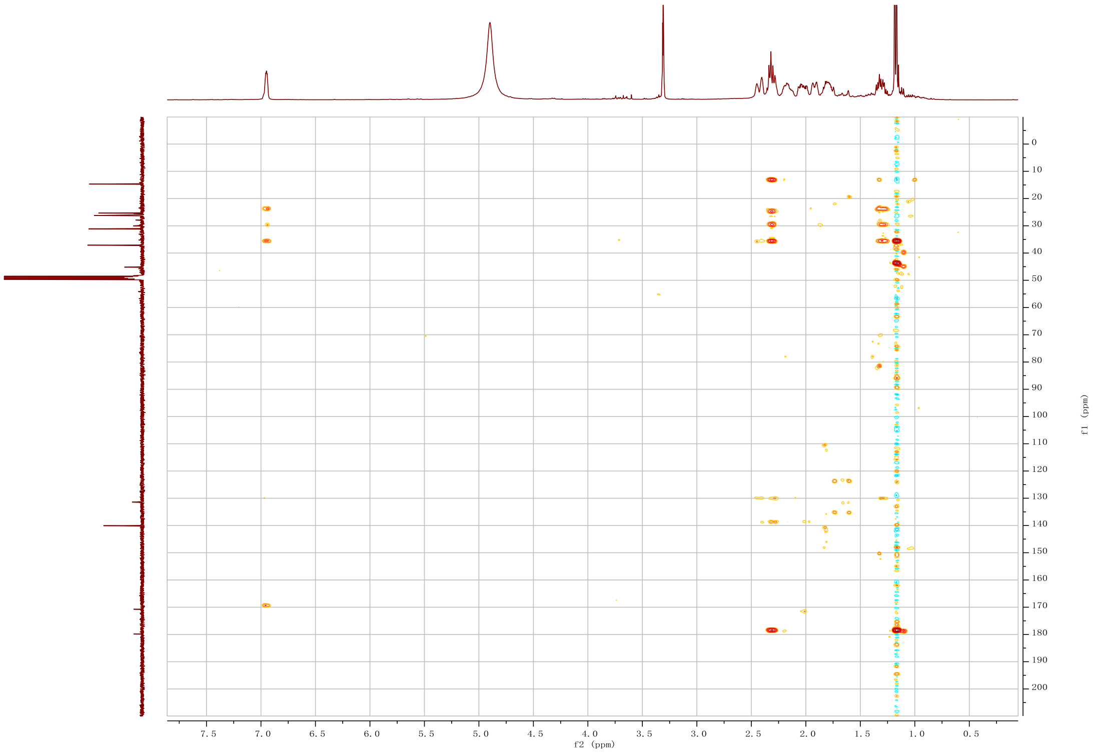

Figure S26. NOESY spectrum of 3 in CD_3OD

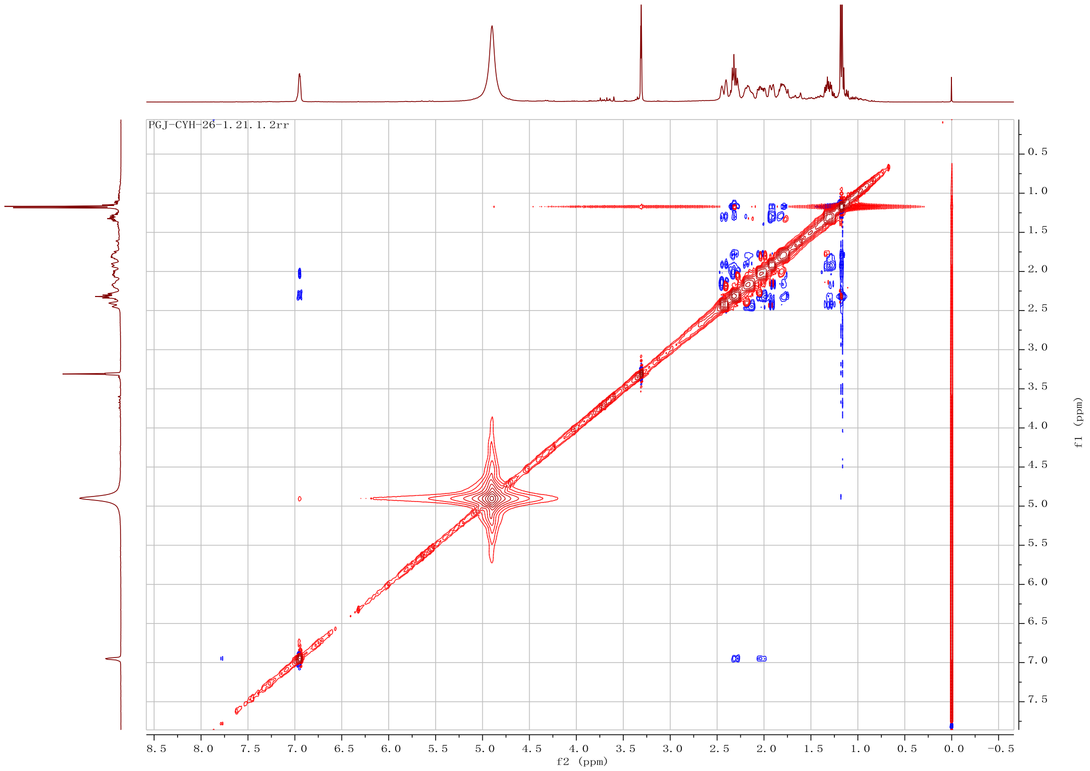

Figure S27. HRESIMS spectrum of 3

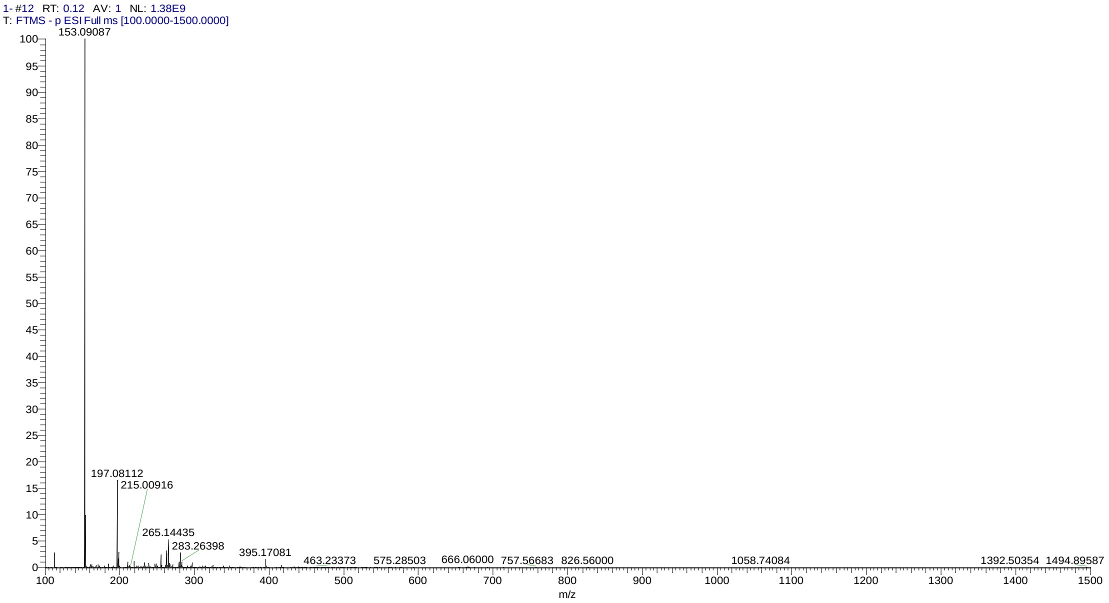

Figure S28. ^{1}H-NMR spectrum of 4 in CD_3OD

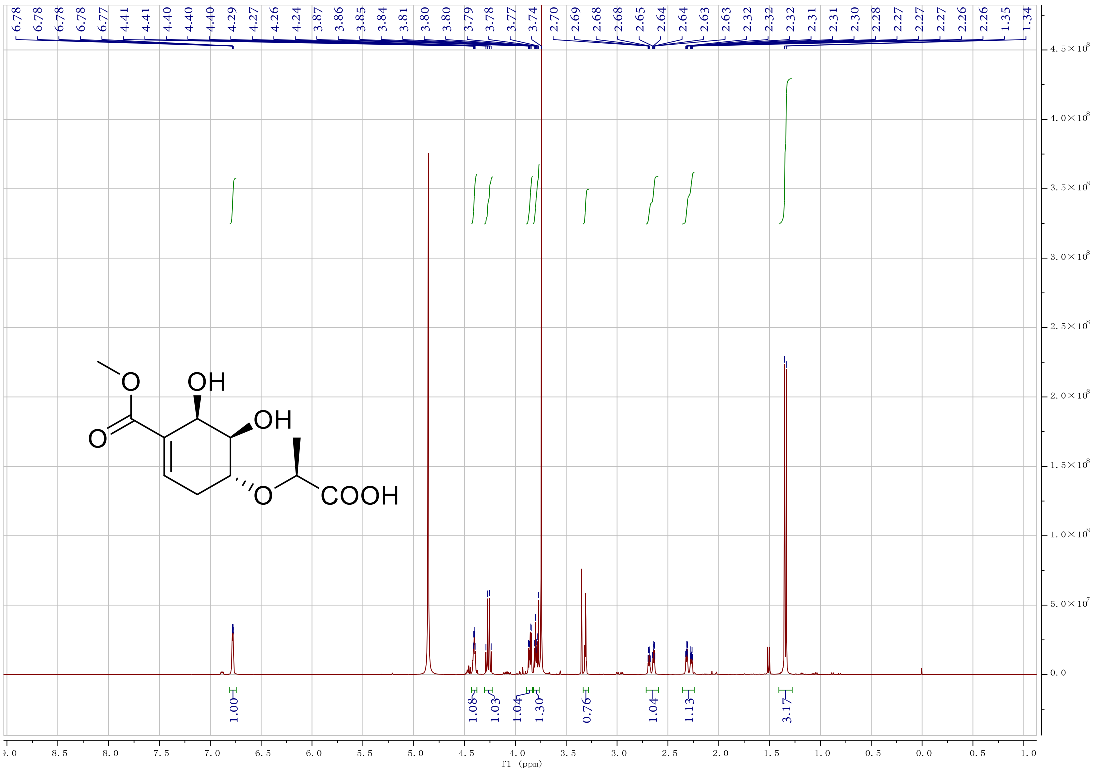

Figure S29. ^{13}C-NMR spectrum of 4 in CD_3OD

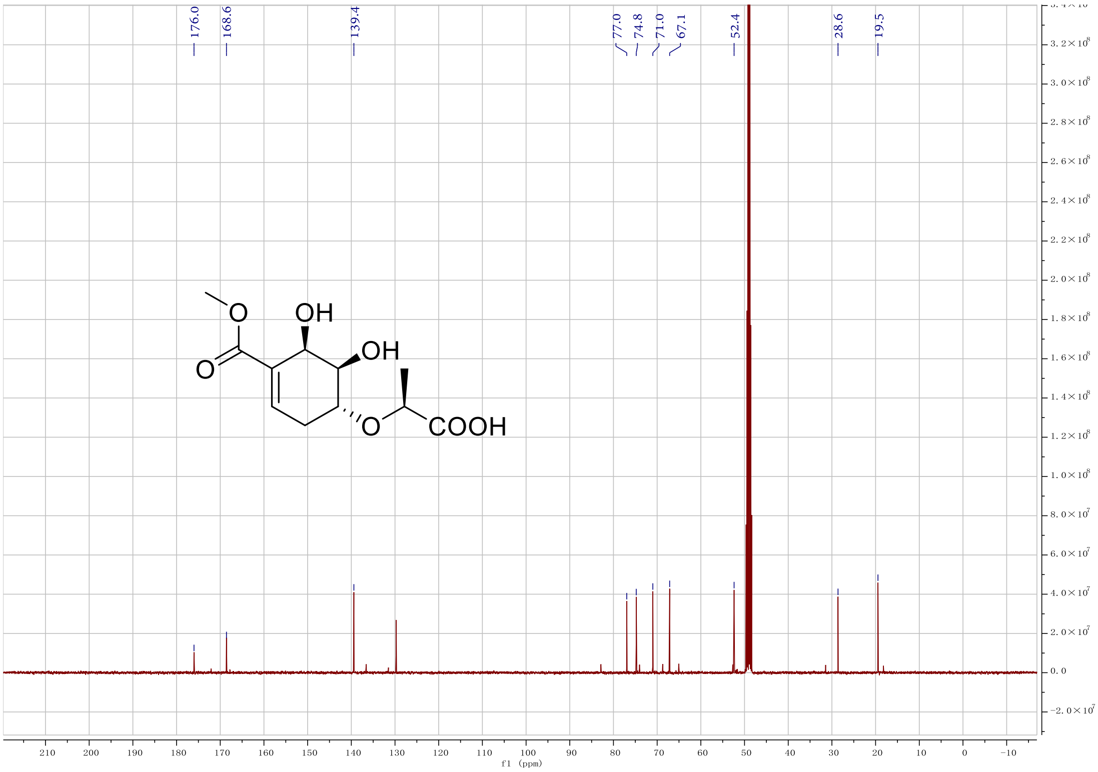

Figure S30. **HMQC spectrum of 4 in CD_3OD**

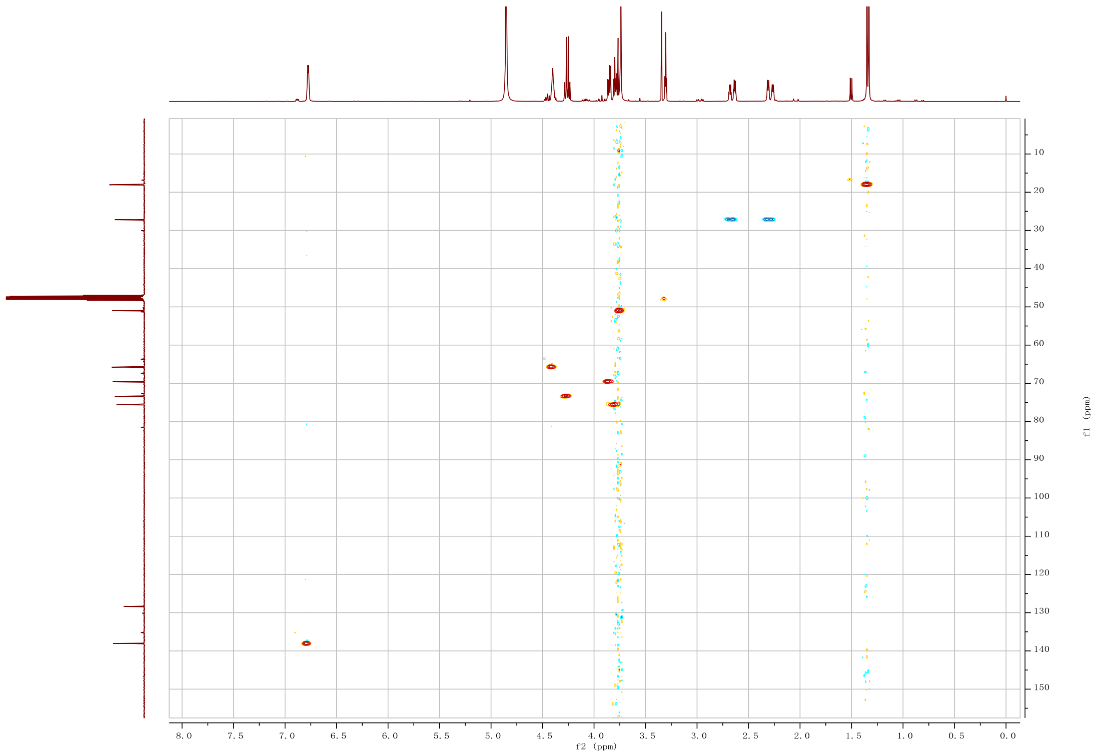

Figure S31. **^1H-^1H COSY spectrum of 4 in CD_3OD**

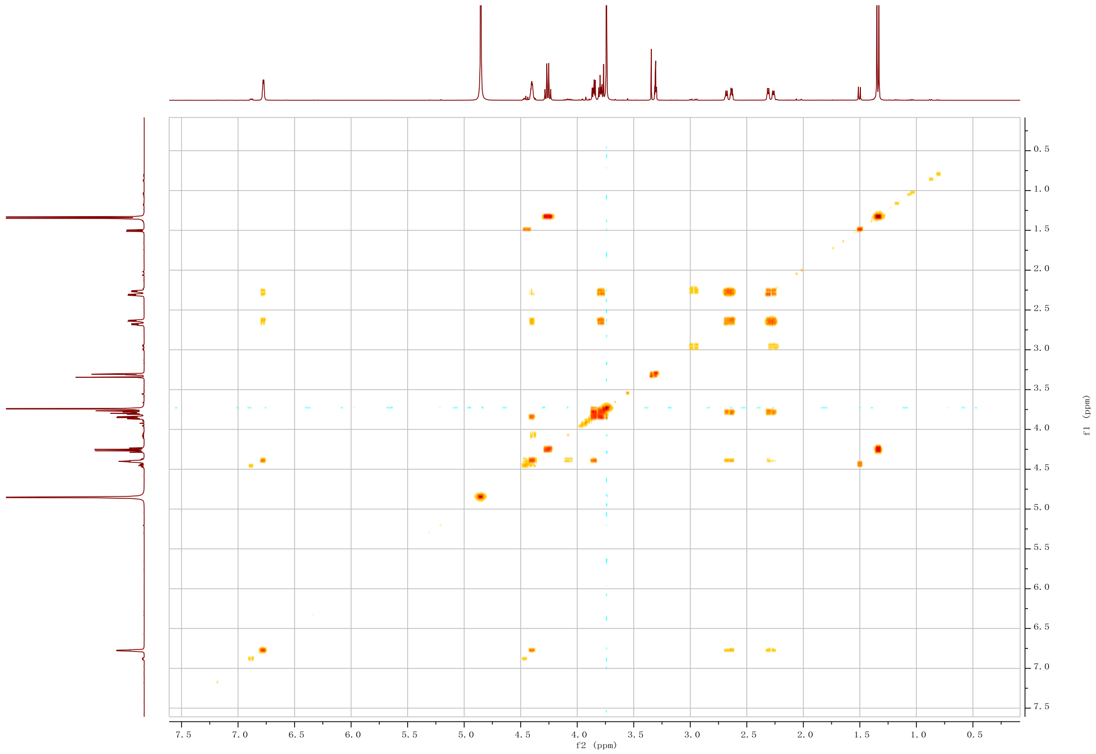

Figure S32. **HMBC spectrum of 4 in CD_3OD**

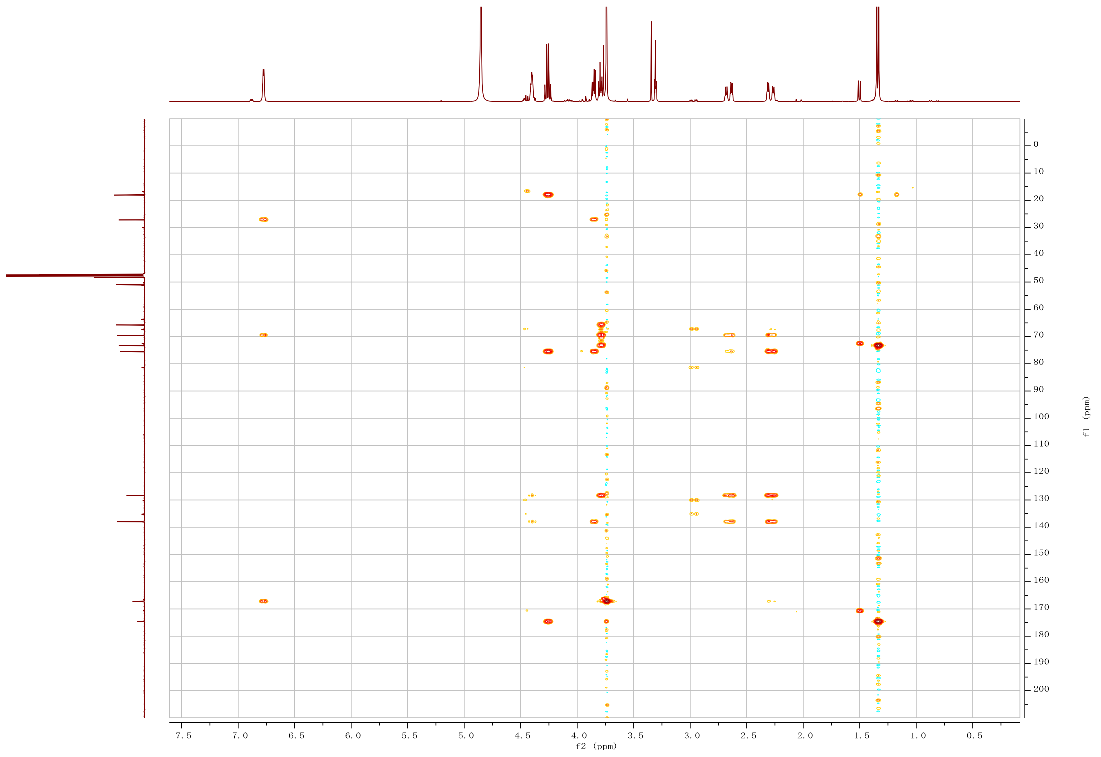

Figure S33. **NOESY spectrum of 4 in CD_3OD**

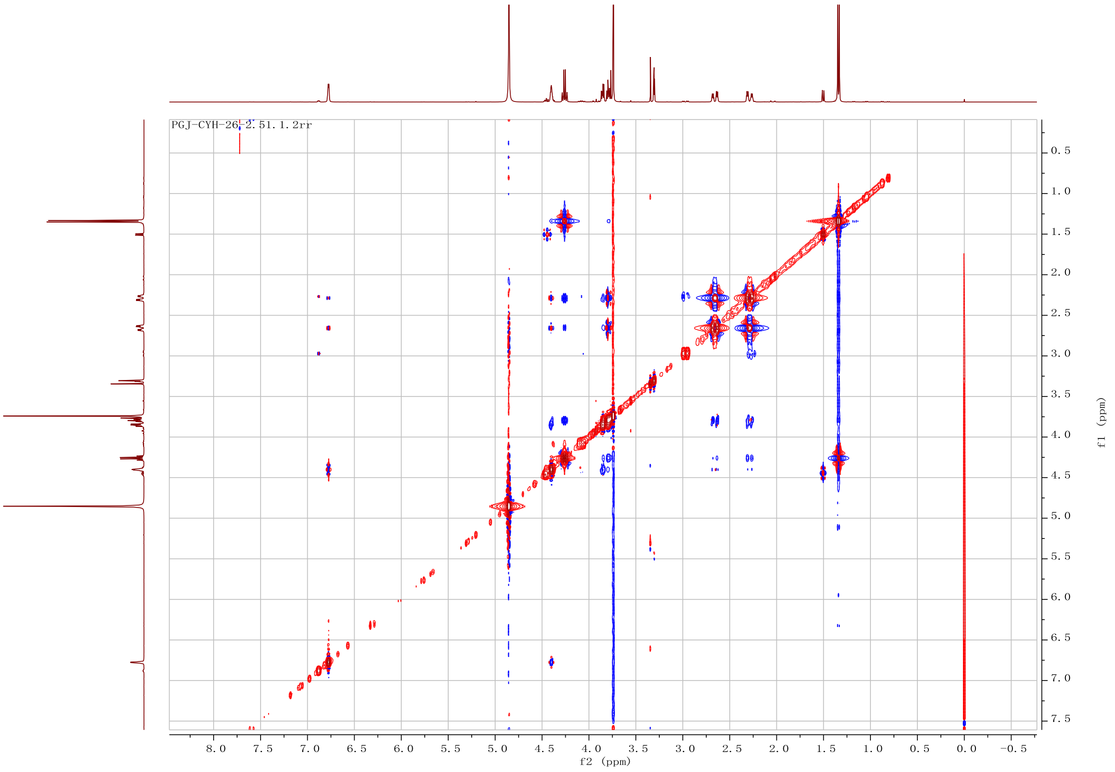

Figure S34. HRESIMS spectrum of 4

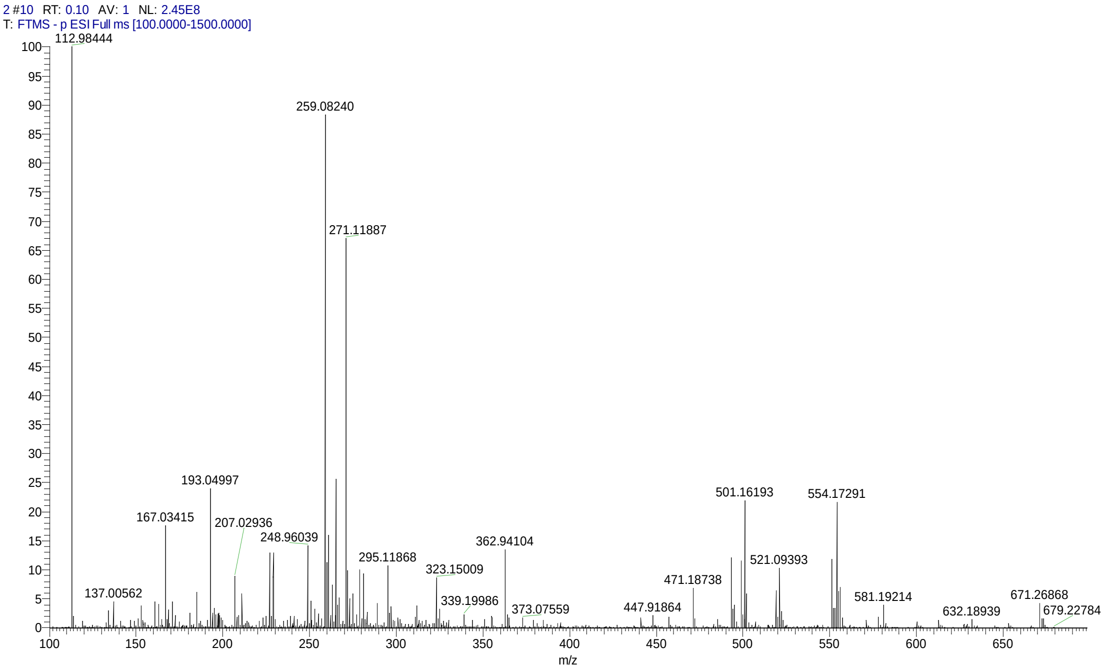

Supplement: Supplementary file 1 [file Data_Sheet_1.PDF]
